# Ecoregion and community structure influences on the foliar elemental niche of balsam fir (*Abies balsamea* (L.) Mill.) and white birch (*Betula papyrifera* Marshall)

**DOI:** 10.1002/ece3.9244

**Published:** 2022-09-11

**Authors:** Travis R. Heckford, Shawn J. Leroux, Eric Vander Wal, Matteo Rizzuto, Juliana Balluffi‐Fry, Isabella C. Richmond, Yolanda F. Wiersma

**Affiliations:** ^1^ British Columbia Government Ministry of Forests, Cariboo Natural Resource Region Williams Lake British Columbia Canada; ^2^ Department of Biology Memorial University of Newfoundland St. John's Newfoundland and Labrador Canada

**Keywords:** biogeography, ecological niche, ecological stoichiometry, intraspecific trait variability, latitudinal patterns, species interactions

## Abstract

Changes in foliar elemental niche properties, defined by axes of carbon (C), nitrogen (N), and phosphorus (P) concentrations, reflect how species allocate resources under different environmental conditions. For instance, elemental niches may differ in response to large‐scale latitudinal temperature and precipitation regimes that occur between ecoregions and small‐scale differences in nutrient dynamics based on species co‐occurrences at a community level. At a species level, we compared foliar elemental niche hypervolumes for balsam fir (*Abies balsamea* (L.) Mill.) and white birch (*Betula papyrifera* Marshall) between a northern and southern ecoregion. At a community level, we grouped our focal species using plot data into conspecific (i.e., only one focal species is present) and heterospecific groups (i.e., both focal species are present) and compared their foliar elemental concentrations under these community conditions across, within, and between these ecoregions. Between ecoregions at the species and community level, we expected niche hypervolumes to be different and driven by regional biophysical effects on foliar N and P concentrations. At the community level, we expected niche hypervolume displacement and expansion patterns for fir and birch, respectively—patterns that reflect their resource strategy. At the species level, foliar elemental niche hypervolumes between ecoregions differed significantly for fir (*F* = 14.591, *p*‐value = .001) and birch (*F* = 75.998, *p*‐value = .001) with higher foliar N and P in the northern ecoregion. At the community level, across ecoregions, the foliar elemental niche hypervolume of birch differed significantly between heterospecific and conspecific groups (*F* = 4.075, *p*‐value = .021) but not for fir. However, both species displayed niche expansion patterns, indicated by niche hypervolume increases of 35.49% for fir and 68.92% for birch. Within the northern ecoregion, heterospecific conditions elicited niche expansion responses, indicated by niche hypervolume increases for fir of 29.04% and birch of 66.48%. In the southern ecoregion, we observed a contraction response for birch (niche hypervolume decreased by 3.66%) and no changes for fir niche hypervolume. Conspecific niche hypervolume comparisons between ecoregions yielded significant differences for fir and birch (*F* = 7.581, *p*‐value = .005 and *F* = 8.038, *p*‐value = .001) as did heterospecific comparisons (*F* = 6.943, *p*‐value = .004, and *F* = 68.702, *p*‐value = .001, respectively). Our results suggest species may exhibit biogeographical specific elemental niches—driven by biophysical differences such as those used to describe ecoregion characteristics. We also demonstrate how a species resource strategy may inform niche shift patterns in response to different community settings. Our study highlights how biogeographical differences may influence foliar elemental traits and how this may link to concepts of ecosystem and landscape functionality.

## Graphical abstract

Foliar elemental niches, defined by axes of carbon (C), nitrogen (N), and phosphorus (P), reveal the ecophysiological responses of species to environmental conditions at regional and local scales. Our study shows that species exhibit biogeographical specific elemental niches likely driven by biophysical ecoregion differences as well as how changes in niche structure occur in response to different community types across scales. Our study highlights biogeographical differences relating to foliar elemental traits linking concepts of ecosystem and landscape ecology.

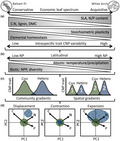

## 1 INTRODUCTION

How we measure and conceptualize a species niche has changed over time. From its original inception of a trait‐habitat match (Grinnell, 1917), our idea of a species niche grew to incorporate species‐environmental feedbacks (Elton, 1927) and their multidimensional resource‐environmental relationships (Hutchinson, 1957). By combining these niche concepts, we can assess species' Intraspecific Trait Variability (ITV) in response to environmental and resource gradients in multidimensional space (Blonder, 2017; Gravel et al., 2019; Soberón, 2007). This approach has provided insights into the structure of food webs (Newsome et al., 2007), foraging behaviors (Hette‐Tronquart, 2019), social interactions (Bergmüller & Taborsky, 2010), community assembly (Bulleri et al., 2016), species networks (Godoy et al., 2018), spatial patterns (Dézerald et al., 2018; Godsoe et al., 2017), and biogeochemical‐environmental relationships (Kearney et al., 2013; Peñuelas et al., 2019; Urbina et al., 2017). However, a potential limitation to comparing niches across different species to reveal environmental relationships is that niche axes, which define a species' ecological role or uniqueness may be constructed using traits which are absent in other species such as differences in root growth patterns; vegetative versus reproductive traits; or trait differences across trophic groups.

Elemental traits represent universal traits to construct niche axes and compare within and between species to reveal how species respond to and exist within variable environments. Although organisms are composed of an elementome of approximately 25 elemental traits (Kaspari & Powers, 2016), carbon (C), nitrogen (N), and phosphorus (P) are the three most proportionately abundant elements (Sterner & Elser, 2002). The concentration of C, N, and P in foliar material provides important linkages to ecological processes (Cherif et al., 2017). For instance, the availability of N and P soil resources regulates C sequestration by influencing an individual's growth and reproductive potential via N and P contributions to enzymes, nucleic acids, and membrane lipids (Elser et al., 2000). Foliar C, N, and P can also indicate nutrient co‐limitation dynamics at the community level where species resource requirements vary in response to competitive effects—adjustments to balance the supply and demand of elemental resources (see Harpole et al., 2011). At broad scales, foliar C, N, and P can be used to infer ecosystem functionality via species‐level elemental plasticity and biogeochemical contributions to nutrient cycling (see Zhang et al., 2018). Recent work highlights the growing interest in using C, N, and P niche axes to assess stoichiometric and trait co‐variability patterns between species, trophic groups, and in response to different environmental conditions (i.e., stoichiometric niche, González et al., 2017; and biogeochemical niche, He et al., 2019; Peñuelas et al., 2019). Thus, foliar C, N, and P represent universal traits to construct niche dimensions and assess ITV that link individuals to environmental conditions across scales such as biogeographical and community‐level gradients (Leal et al., 2017).

Plants are distributed across biogeographic gradients and likely alter their resource strategies (resource acquisition and use) in response to differing biophysical constraints of temperature, precipitation, and soil nutrient/moisture regimes (Šímová et al., 2011). For instance, the temperature‐plant physiological hypothesis suggests plants at higher latitudes contain greater foliar N and P elemental concentrations (Reich & Oleksyn, 2004). This is attributed to lower photosynthetic gains of C in colder temperatures relative to N and P uptake (Woods et al., 2003). As well, low foliar P can indicate stressful environmental conditions species might experience on the edge of their range, such as drought (He et al., 2019). Moreover, by evaluating foliar elements along niche axes, we can link changes in C, N, and P relationships via ITV and trait co‐variability patterns to broad biogeographical environmental classification schemas (i.e., ecozone, ecoregion, and ecodistrict), and their associated biophysical and climate factors to better understand top‐down controls on species ecophysiology (Ecological Stratification Working Group, 1996; MacKenzie & Meidinger, 2018).

Across, within, and between biogeographical areas, trees often occur in spatial associations of conspecific and heterospecific communities (i.e., trees in pure and mixed wood forest stands; Hansson, 1992; Pastor et al., 1999). In these communities, differing mechanisms of dispersal, nutrient use, herbivory, and disturbance interact to influence the recruitment of juvenile trees that will eventually replace adults (Birch et al., 2019; Gray & He, 2009). As stands develop, horizontal and vertical community structure differs, and this can influence the presence and abundance of recruiting individuals via light availability and litter‐biochemical soil interactions (Klinka et al., 1996). In conspecific and heterospecific communities, variability in community structure can arise from differing types (i.e., needleleaf and broadleaf), amounts, and chemical compositions (i.e., low C:N) of foliar litter input (Gartner & Cardon, 2004; Hobbie, 2015). This in turn influences microbial community composition and regulates decomposition and nutrient recycling processes (Krishna & Mohan, 2017; Prieto et al., 2019). For example, in conspecific communities, positive feedbacks have been observed for biogeochemical processes of nutrient recycling via nutrient retrieval (Florence & McGuire, 2020). In comparison, heterospecific associations often promote diversification of microbial communities in response to differing types of litter input, which in turn increases the competition for nutrient retrieval (Krishna & Mohan, 2017; Reynolds et al., 2003). Thus, trees in conspecific and heterospecific communities experience different community structural and nutrient feedback conditions that regulate N and P uptake and C sequestration and this is reflected in foliar C, N, and P concentrations (Reich et al., 2009; Urbina et al., 2017).

Recent work demonstrates the linkages of foliar elemental niche patterns to different community types. For example, Urbina et al. (2017) characterized biogeochemical niche hypervolume shifts as either an expansion, contraction, or displacement responses relative to a conspecific niche (i.e., community occurrence of the same species) using a principal component analysis. As well, different niches can be compared by assessing hypervolume patterns of niche similarity via size, overlap, and nestedness (for Jaccard hypervolume comparisons see Blonder et al., 2014). For instance, González et al. (2017) constructed niche hypervolumes centered around averaged stoichiometric coordinates and compared how these niche hypervolumes differ in shape, size, and location. This allowed them to reveal intraspecific trait variability across plants, invertebrates, and vertebrates. These examples demonstrate approaches to compare how the elemental niches of species may differ across biogeographic regions and in response to different community compositions such as when they occur in groups of the same species (i.e., conspecific) and when they co‐occur in groups of mixed species (i.e., heterospecific).

Framing species by their resource strategies in terms of how they acquire and use C, N and P provides a link to compare and contrast species elemental niches in response to different environmental conditions. Conceptually, C, N, and P likely differ among plant species along a spectrum of conservative to acquisitive resource strategies (Craine, 2005). These strategies describe how species make different resource acquisition and use trade‐offs to optimize performance in variable environments. Moreover, species with different resource strategies often require different elemental concentrations (i.e., homeostasis for proper physiological function) and exhibit different stoichiometric plasticity (variability of elemental ratios) related to environmental conditions (Fajardo & Siefert, 2018; Leal et al., 2017; Stearns, 1989). For instance, coniferous species with conservative resource strategies produce long‐lived needles and often exhibit low needle morphological variability and limited foliar growth geometry (Horn, 1971). Thus, conifers tend to have a high elemental homeostasis and low stoichiometric plasticity where foliar C, N, and P concentrations are constrained by a narrow range of eco‐physiological conditions (Marshall & Monserud, 2003). In comparison, fast‐growing, shade‐intolerant deciduous species with acquisitive resource strategies, such as those that produce and shed seasonal foliar material, often display low elemental homeostasis and high stoichiometric plasticity via variable leaf morphology; and hence, more flexibility in how they use N and P resources (Middleton et al., 1997). By linking species resource strategies to their elemental homeostasis and stoichiometric plasticity, we can compare and contrast foliar elemental niche differences across biogeographic gradients and in response to different community compositions to reveal species‐trait generalities at large and local spatial extents.

**FIGURE 1:**
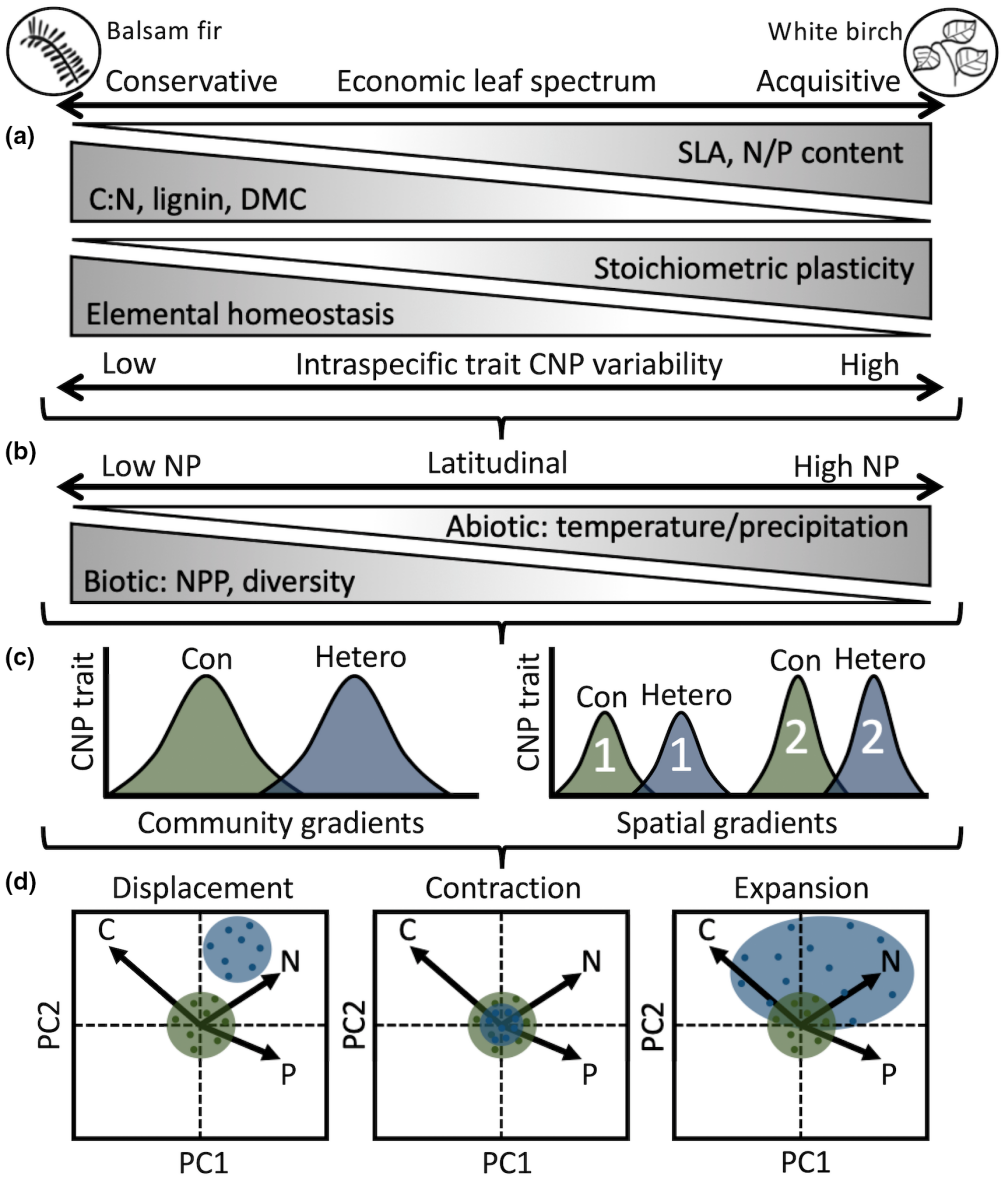
Conceptual diagram for foliar elemental niche differences. Our two focal species, balsam fir and white birch are depicted at the top of the diagram operating on different ends of a resource strategy. Here, differences in conservative and acquisitive foliar traits are related to life history strategies of resource acquisition, use, and storage (a). For instance, slow‐growing conservative species which produce long‐lived foliar material often exhibit high foliar C:N, lignin, and dry matter content (DMC) as durable foliar traits as opposed to traits of high specific leaf area (SLA) and N/P concentration for fast‐growing acquisitive species, which shed foliar material annually. Although we highlight other foliar traits in this diagram, our study focuses on foliar elemental traits of C, N, and P as they relate to elemental homeostasis and stoichiometric plasticity. Due to resource acquisition and use tactics, conservative species often exhibit low stoichiometric plasticity and high elemental homeostasis as compared to the high stoichiometric plasticity and low elemental homeostasis of acquisitive species. Furthermore, internal elemental demands and eco‐physiological constraints limit the intraspecific trait variability (ITV) of foliar C, N, and P. And as a filter for community assembly, traits and their intraspecific variability are used to explain niche mechanisms of biotic interactions such as trait conditions under different community settings. As well, latitudinal patterns of foliar N and P are often associated with gradients of temperature and precipitation with lower photosynthetic gains of C in colder temperatures relative to N and P uptake (b). The assertion that biological diversity and Net Primary Production (NPP) decrease with distance from the tropics is associated with intensified competitive interactions that may reduce resource availability. This suggests that populations in northern biogeographic locations should have higher foliar N and P concentrations relative to southern populations. Here, we use foliar C, N, and P traits as it relates directly to resource use and niche mechanisms to assess how the elemental niche of balsam fir and white birch differs at a species level and community level. At the species level (c), we expect both our focal species to exhibit larger elemental niche volumes in our northern ecoregion (Northern Peninsula) compared to their niche volumes in our southern ecoregion (Central Forest). At a community level, we assess the niche hypervolumes of our focal species by conspecific (only one focal species present) and heterospecific (both focal species present) groups. We expect their elemental niche hypervolumes to be different when in a conspecific (Con; green) as opposed to a heterospecific (Hetero; blue) community types (c). We make these community‐level comparisons across, within, and between ecoregions. For across‐ and within‐ecoregion comparisons, we expect balsam fir heterospecific niche hypervolumes to be displaced relative to the conspecific niche hypervolume and for white birch we expect a displacement pattern (d)—these potential patterns reflect their resource strategies, elemental homeostasis, and stoichiometric plasticity described above via principal component analysis (PCA; Peñuelas et al., 2019; Urbina et al., 2017). For between‐ecoregion comparisons, we expect niches in the northern ecoregion to be larger in volume relative to their corresponding niche (i.e., conspecific vs. conspecific) in the southern ecoregion for both balsam fir and white birch.

Here, we construct niche hypervolumes using axes of foliar C, N, and P traits for balsam fir (*Abies balsamea* (L.) Mill.) and white birch (*Betula papyrifera* Marshall), two widespread North American boreal forest species. These focal species exhibit different resource strategies (i.e., coniferous and deciduous) and have contrasting foliar elemental homeostasis and stoichiometric plasticity characteristics that may be influenced by large‐scale (i.e., ecoregion biophysical conditions) and small‐scale (i.e., community‐level dynamics) processes (Hausch et al., 2018; Richardson, 2004). First, we investigate elemental niche differences at the species level. Second, at a community level, we compare heterospecific (i.e., both focal species present) against conspecific niches (i.e., only one focal species present) and determine relative niche hypervolume shift patterns across, within, and between ecoregions. At the species level, we hypothesize (H1) that the northern ecoregion foliar elemental niche for each of focal species will be larger in volume relative to their southern ecoregion niche, driven by increased foliar N and P concentrations that follows the temperature‐plant physiology hypothesis (see Reich & Oleksyn, 2004). At the community level, we first compare conspecific (i.e., reference niche) against heterospecific niches across ecoregions (i.e., irrespective of ecoregion) and we hypothesize (H2) that balsam fir, given limited foliar stoichiometric plasticity, will exhibit a niche hypervolume displacement pattern, where the proportionality of foliar elements remains similar but the two niches occupy different space. In comparison, we hypothesize (H3) that white birch, given a high degree of stoichiometric plasticity, will exhibit a niche hypervolume expansion pattern where heterospecific conditions increase variability of foliar elemental traits and thus increased niche hypervolume relative to the conspecific niche. For within‐ecoregion comparisons (i.e., conspecific vs. heterospecific within an ecoregion), again, we expect species niche patterns to reflect their resource strategy, and we hypothesize niche hypervolume displacement for balsam fir (H4) and niche hypervolume expansion (H5) for white birch. For between‐ecoregion comparisons (e.g., conspecific vs. conspecific between ecoregion), for both balsam fir and white birch we hypothesize (H6) that conspecific and heterospecific northern ecoregion niches will be larger in volume relative to their respective southern ecoregion niche hypervolume (see Figure 1 for a conceptual description of our hypotheses and Table 1 for a summary of the hypotheses described above).

**TABLE 1:** Summary of hypotheses and expected results for each of our comparisons.

Hypothesis	Description	Expectation
H1	Balsam fir and white birch, comparing north versus south ecoregion niche hypervolumes	Species niches in the northern ecoregion will be larger in volume relative to their southern ecoregion niche
H2	Balsam fir, comparing conspecific versus heterospecific niche hypervolumes	Heterospecific niche displacement relative to the conspecific niche hypervolume
H3	White birch, comparing conspecific versus heterospecific niche hypervolumes	Heterospecific niche expansion relative to the conspecific niche hypervolume
H4	Balsam fir, comparing conspecific versus heterospecific niche hypervolumes within north and south ecoregions	In both ecoregions, heterospecific niche displacement relative to the conspecific niche hypervolume
H5	White birch, comparing conspecific versus heterospecific niche hypervolumes within north and south ecoregions	In both ecoregions, heterospecific niche expansion relative to the conspecific niche hypervolume
H6	Balsam fir and white birch, comparing conspecific versus conspecific and heterospecific versus heterospecific niche hypervolumes between north and south ecoregions	Species conspecific and heterospecific niches in the northern ecoregion will be larger in volume relative to their corresponding conspecific and heterospecific niche in the southern ecoregion

## 2 MATERIALS AND METHODS

### 2.1 Study area

Our study areas consist of two ecoregions on the island of Newfoundland: (1) the Northern Peninsula and (2) the Central Newfoundland forest ecoregions (see Appendix S1: Figure S1 for a study area map). Ecoregions are distinct areas characterized by major physiographic and minor macroclimatic differences, including vegetative, soil, water, fauna, and land‐use differences (Ecological Stratification Working Group, 1996). Our ecoregions and corresponding sampling sites are approximately two latitudinal degrees apart (a 300 km distance). The Northern Peninsula ecoregion has a mean annual temperature of 3°C, with mean summer and winter temperatures of 11 and −4.5°C, respectively, and a mean annual precipitation of 1000–1100 mm. Balsam fir is the dominant tree species in this ecoregion on well‐to‐moderately drained sites, whereas black spruce (*Picea mariana* (Mill.) Britton, Sterns, & Poggenb) and white birch are important co‐dominant species. The soil type is generally humo‐ferric podzols (South, 1983). The Central Newfoundland Forest ecoregion (hereafter referred to as Central Forest ecoregion) has a mean annual temperature of 4.5°C, with mean summer and winter temperatures of 12.5 and −3.5°C, respectively, and a mean annual precipitation of 1000–1300 mm. The forests of this ecoregion are dominated by closed stands of balsam fir with co‐dominants of white birch, black spruce, trembling aspen (*Populus tremuloides* Michx.), and eastern larch (*Larix laricina* (Du Roi) K. Koch). Generally, the soil type is humo‐ferric podzols with gleyed podzols and brunisolic and gleysolic soils. These two ecoregions also differ in terms of shoulder season temperature and precipitation, soil‐topographic relationships, and historical disturbance patterns (e.g., insect outbreak, wind, and fire; Arsenault et al., 2016; South, 1983).

### 2.2 Plant sampling

During the summer months, June to August, we collected samples of balsam fir and white birch from the Northern Peninsula ecoregion in 2015 and Central Forest ecoregion in 2016. Samples consisted of the forage material from juvenile trees (i.e., foliage and incidental woody bits) between 0 and 2 m in height, the vertical range commonly used by moose (*Alces alces* (Linnaeus, 1758)) and snowshoe hare (*Lepus americanus* (Erxleben, 1777)). The variability of foliar elemental traits likely influences animal space‐use decisions, and this study is part of a larger research project focused on understanding elemental‐trophic linkages (see Balluffi‐Fry et al., 2021; Rizzuto et al., 2021). As well, we collected samples from a variety of stand types under the canopy and are representative of various canopy closure conditions. Although the sampling design differed between 2015 and 2016 in terms of plot size (2015 and 2016 plot radii were 10 and 11.3 m, respectively) and the spatial arrangement of plots, the sampling units of C, N, and P are the same. More specifically, in 2015, we randomly placed sample plots stratified by forest age within different forest types (coniferous, deciduous, and mixedwood) at a minimum of 500 m apart and within 200 m of resource roads. In 2016, we set up four grids stratified by forest age and dominant forest type and sampled along meandering transects at each grid with plots spaced 75 m apart and 37 m spacing at grid corners. At the plot level, we collected samples in both 2015 and 2016 in the same manner. We divided each sample plot into four quadrants, and if present, we collected foliage from balsam fir and white birch individuals in each quadrant. We moved clockwise between each quadrant and collected foliage until a suitable amount of wet weight was collected (approx. 10 g). Lastly, we combined foliage samples from individuals by species per plot using representative foliage material until we achieved a mass suitable to determine C, N, and P concentrations (approx. 10 g; Northern Peninsula data used in this study are from Leroux et al., 2017).

Since we used data collected from Leroux et al. (2017) in an ad hoc opportunity, the sampling design is unbalanced between the two ecoregions being compared. In total, we had 390 balsam fir and 229 white birch samples. For our species‐level comparisons between ecoregions, we had 295 Northern Peninsula and 95 Central Forest samples of balsam fir and 158 Northern Peninsula and 71 Central Forest samples of white birch (i.e., the *n* used to test H1). At the plot level, we determined the conspecific or heterospecific conditions based on the presence/absence of either balsam fir or white birch. For instance, a plot was considered conspecific if it only had one of the focal species present and heterospecific if it had both focal species present. For our community‐level comparisons across ecoregions, we had 189 conspecific and 201 heterospecific samples of balsam fir (i.e., the *n* used to test H2) and 28 conspecific versus 201 heterospecific samples of white birch (i.e., the *n* used to test H3). For our community‐level comparisons of balsam fir within and between ecoregions, we had 142 conspecific and 153 heterospecific samples in the Northern Peninsula ecoregion and 47 conspecific and 48 heterospecific samples in the Central Forest ecoregion (i.e., the *n* used to test H4/H6). For our community‐level comparisons of white birch within and between ecoregions, we had 5 conspecific and 153 heterospecific samples in the Northern Peninsula ecoregion and 23 conspecific and 48 heterospecific samples in the Central Forest ecoregion (i.e., the *n* used to test H5/H6).

### 2.3 Lab analysis

Foliage samples were processed by the Agriculture Food Lab (AFL) at the University of Guelph. Total C and N concentration (as % dry weight) was determined using an Elementar Vario Macro Cube. Total P concentration (as % dry weight) was determined using a microwave acid digestion CEM MARSxpress microwave system and brought to volume using Nanopure water. The clear extract supernatant was further diluted by 10 to accurately fall within calibration range and reduce high‐level analyte concentration entering the inductively coupled plasma mass spectrometry detector (ICP‐MS; see Poitevin, 2016).

### 2.4 Analysis and interpretation

For each of our focal species, we make four comparisons. At the species level, we compare foliar elemental niche hypervolumes across ecoregions (i.e., Northern Peninsula compared against the Central Forest niche as the reference point; H1). We then compare niche hypervolume community types of heterospecific groups against and conspecific groups (i.e., reference point), across (H2/H3), within (H4/H5), and between (H6) ecoregions. For each comparison, we performed several different analyses to characterize and assess niche differences. Using the factoextra R package, we performed a PCA to characterize niche hypervolume response patterns as either a displacement, contraction, or expansion via the position, shape, and size of the two 95% probability ellipses relative to each other and quantified using additional measures described below (Peñuelas et al., 2019; Urbina et al., 2017). Using the vegan R package (Oksanen et al., 2020), we computed the multivariate homogeneity of variances (MHD) for niche hypervolume spatial median/centroid. Using these data, we computed a permutation test for homogeneity of multivariate dispersion (PT‐MHD) and report the *F* value and *p*‐value for 999 permutations. This test permutes model residuals and generates the distribution of *F* for a null hypothesis where no difference in dispersion exists. If the *p*‐value from the PT‐MHD test is significant, then heterogeneity in dispersion exists. The PT‐MHD test is useful for assessing bias when comparing groups with unequal size sample. PERMANOVA tests are sensitive to unequal sample sizes and require groups to exhibit homogeneous dispersion (Anderson, 2006). We use 999 permutations and Bray–Curtis distances to calculate pairwise comparisons of niche hypervolumes and report *R* ^2^, *F* statistic, and *p*‐value PERMANOVA results. For each PERMANOVA comparison, significant niche hypervolume differences occur when *p*‐value ≤ .05 (see Appendix S2: Table S1 for full PERMANOVA results). In addition, we used the hypervolume R package (Blonder et al., 2014), to construct hypervolumes for each niche based on Gaussian kernel density estimation with a probability density enclosed by a 95% probability boundary. Using these hypervolume niche comparisons, we report the Jaccard similarity index to aid in our interpretation of niche differences (Blonder, 2017).

Using publicly accessible code from González et al. (2017), we evaluated niche volume, overlap, nestedness, shape, and assessed for sample size effects given the number of individuals in our ecoregion and community type groupings (see Appendix S3: Figure S2). Niche size/volume, a convex hull calculation, represents variability of C, N, and P or ITV. Niche overlap is then the ratio of shared volume between each niche, presented as a percentage (i.e., the sum of two volumes minus the intersecting volume). The degree of niche hypervolume overlap indicates the similarity or difference of C, N, and P traits between them. Moreover, niche hypervolume nestedness represents the extent of niche overlap, using the ratio of the overlapping niche volume relative to the minimal volume occupied to produce a value on a scale of 0–1, with 0 indicating no nestedness and 1 indicating complete nestedness. Niche overlap and nestedness metrics describe niche position and size between groups. Niche nestedness helps to discriminate between different niche overlap patterns, such as overlap when sharing a similar proportion of niche volume and overlap when one niche occupies a subset of another niche volume. Lastly, we assess for sample size effects on niche hypervolumes using representative subsampling approach as opposed to rarefaction, which has been shown to potentially underestimate the hypothetical true niche hypervolume for uncommon or less abundant species (González et al., 2017; Willis, 2019). Following González et al. (2017), we subsampled an increasing number of individuals at specified intervals depending on the number of samples we had for a given niche hypervolume. For each interval, we calculated niche hypervolumes using 999 randomized permutations and quantified variability using 95% confidence intervals and continued until all individuals were sampled for each niche hypervolume (see Appendix S3: Figure S2).

We determined ITV responses for each of our focal species comparisons by subtracting niche hypervolumes against each other using Central Forest or conspecific (i.e., for between‐ecoregion comparisons) niche hypervolumes as reference points. For between‐ecoregion comparisons, we subtracted Central Forest niche hypervolumes from Northern Peninsula niche hypervolumes. For across‐ecoregion comparisons, we subtracted conspecific niche hypervolumes from heterospecific niche hypervolumes. For within‐ecoregion comparisons, we subtracted conspecific niche hypervolumes from heterospecific niche hypervolumes for each ecoregion. For between‐ecoregion comparisons, we subtracted Central Forest niche hypervolumes of conspecific and heterospecific against their corresponding community type niche hypervolume in the Northern Peninsula ecoregion. Lastly, we assessed latitudinal patterns by subtracting foliar C, N, and P means.

We depicted niche hypervolumes in three‐dimensional data space, we use spherical representations centered around the averaged C, N, and P coordinates as opposed to polygonal features, where many edges, vertices, and faces make it difficult to visually discern general patterns (González et al., 2017). See Appendix S4: Table S2 for each niche sample size, Shapiro–Wilk test of multivariate normality for each niche, and volume as determined using niche metrics from González et al., 2017.

## 3 RESULTS

### 3.1 Sample size effects

Our representative subsampling analysis to evaluate sample size effects on niche hypervolume demonstrates potential limitations for small sample sizes for some comparisons. In Appendix S3: Figure S2, we show mean niche hypervolume curves with increasing sample size until all individuals have been sampled. In most cases, variation in the relationship between niche hypervolume and sample size decreased with sample size and tend toward an asymptote, indicating sample saturation. Where subsampling results do not reach an asymptote (i.e., sample saturation does not occur), we have limited confidence where these niche hypervolumes are used in species and community‐level comparisons. More specifically, the less reliable niche hypervolume comparisons include the following: white birch conspecific across ecoregion (*n* = 28), Northern Peninsula conspecific (*n* = 5), and Central Forest conspecific (*n* = 23) and heterospecific niches (*n* = 48), which do not appear to reach an asymptote when plotting niche hypervolume against sample size (see Appendix S3: Figure S2). These four niches impact five out of our six comparisons for white birch (i.e., all the community‐level results are less reliable, thus, only the species‐level comparison is reliable).

### 3.2 Species level: between ecoregions

**FIGURE 2:**
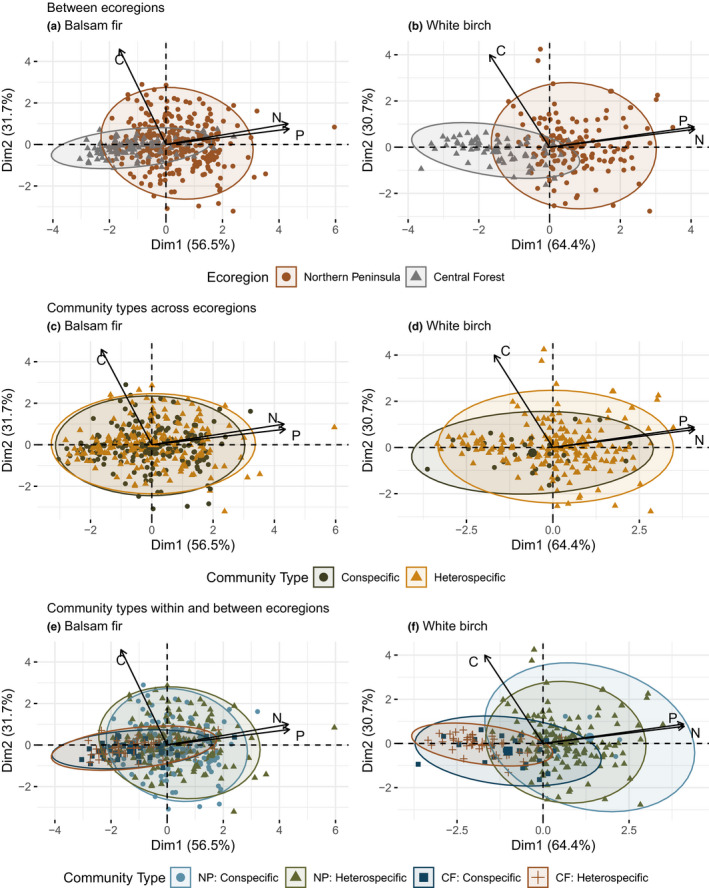
Principal component analysis (PCA) for balsam fir (a) and white birch (b) at the species‐level between ecoregions and at the community level across (c and d), within, and between (e and f) ecoregions. For each plot, ellipses with a 95% probability are shown for each comparison and color‐coded for ecoregions (a and b), conspecific and heterospecific groups (c and d), and conspecific and heterospecific groups by ecoregions (e and f). In addition, different symbology is used in these plots to showcase the variability of individuals of different niches. In both cases, dimension 1 explains 56.5% and 64.4% while dimension 2 explains 31.7% and 30.7% of the variance for balsam fir and white birch, respectively. In all cases, N and P highly influence dimension 1 while C influences dimension 2.

**FIGURE 3:**
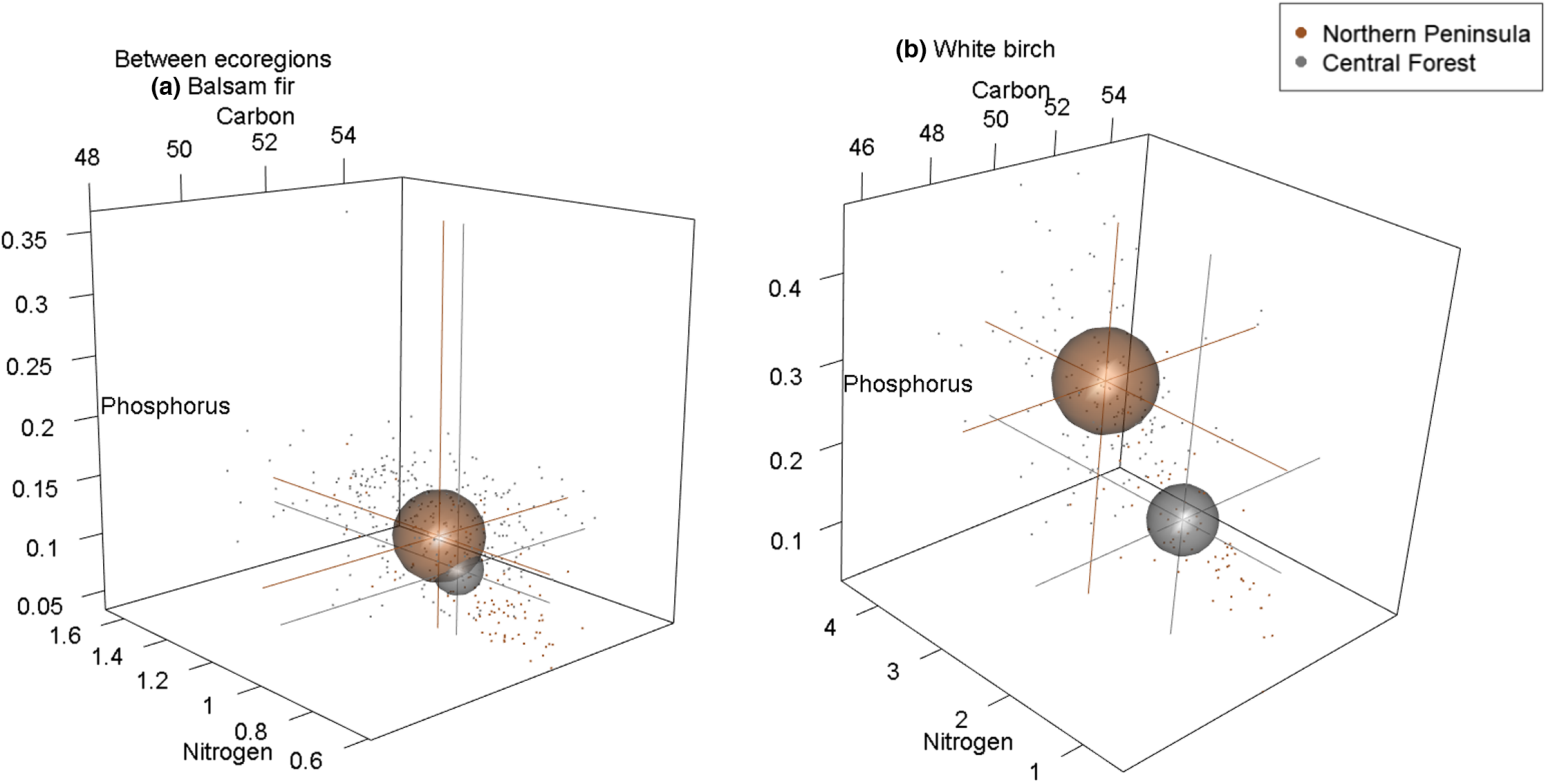
Spherical representations of niche hypervolumes at the species level for between‐ecoregion comparisons for balsam fir (a) and white birch (b). Plot size represents the total stoichiometric volume of C, N, and P for each focal species. Corresponding drop lines to axes indicate the average C, N, and P value for each niche.

Our hypothesis for both balsam fir and white birch that elemental niches for individuals from the northern ecoregion will be larger in volume relative to their southern ecoregion niche is supported by our results (H1). Our PCA reveals individuals from the Northern Peninsula ecoregion occupy larger foliar elemental trait space compared with individuals from the Central Forest ecoregion (Figure 2a,b). For balsam fir, variance explained by axes 1 and 2 is 56.5% and 31.7%, respectively (Figure 2a) and for white birch variance explained by axes 1 and 2 is 64.4% and 30.7%, respectively (Figure 2b). PERMANOVA results indicate significant differences between Northern Peninsula and Central Forest elemental niche hypervolumes for balsam fir (*F* = 14.592, *p‐*value = .001) and white birch (*F* = 75.999, *p‐*value = .001; see Table 2). However, our permutation test for homogeneity of multivariate dispersion (PT‐MHD) was significant for both balsam fir (*F* = 57.683, *p‐*value = .001) and white birch (*F* = 9.174, *p‐*value = .005); as an assumption for PERMANOVAs, this potentially limits our interpretation (Table 2). The Jaccard similarity index indicates a low degree of niche hypervolume similarity between Northern Peninsula and Central Forest niches for balsam fir (0.281) and white birch (0.163; see Table 2). For balsam fir, our niche volume metrics indicate low overlap (10.714%), moderate nestedness (0.393), and increased ITV via niche volume (+70.97%) for the Northern Peninsula niche (see Figure 3a). For white birch, niche volume metrics indicate a low overlap (5.166%), low nestedness (0.067), and increased ITV (+46.65%) via niche volume for the Northern Peninsula niche (see Figure 3b and Table 2). Lastly, foliar N and P were greater for the Northern Peninsula ecoregion for balsam fir by a difference of 0.164% and 0.049% and for white birch, 1.143% and 0.127%, respectively. For balsam fir and white birch, foliar C was greater in the Central Forest ecoregion by 0.205% and 0.545%, respectively (Table 3a). In addition, these results are supported by our niche sample size analysis as all four niche hypervolumes used in these comparisons are likely of sufficient sample size (Appendix S3: Figure S2). As well, see Appendix S5: Figure S5 for a pairwise scatter plot comparison of foliar C, N, and P between ecoregions for balsam fir and white birch.

**TABLE 2:** Summary of niche comparison results for balsam fir and white birch.

Balsam fir	MHD		PT‐MHD		PERMANOVA			Hypervolume	Niche volume metrics		
Between ecoregion	**NP**	**CF**	*F* value	*p*‐Value	*R* ^2^	*F* statistic	*p*‐Value	Jaccard	Overlap	Nestedness	ITV (%)
	0.011	0.005	57.683	**.001**	.036	14.592	**.001**	0.281	10.714%	0.393	+70.97
CT: across ecoregion	**Con**	**Hetero**	*F* value	*p*‐Value	*R* ^2^	*F* statistic	*p*‐Value	Jaccard	Overlap	Nestedness	ITV
	0.009	0.010	0.065	.805	.002	0.646	.458	0.709	43.860%	0.276	+35.49
CT: within ecoregion	**Con**	**Hetero**	*F* value	*p*‐Value	*R* ^2^	*F* statistic	*p*‐Value	Jaccard	Overlap	Nestedness	ITV
Northern Peninsula	0.011	0.011	0.140	.716	.002	0.450	.570	0.672	40.426%	0.251	+29.04
Central Forest	0.005	0.005	0.051	.836	.003	0.306	.726	0.566	50.000%	0.000	0.000
CT: between ecoregion	**NP**	**CF**	*F* value	*p*‐Value	*R* ^2^	*F* statistic	*p*‐Value	Jaccard	Overlap	Nestedness	ITV
Conspecific	0.011	0.005	25.902	**.001**	.039	7.581	**.005**	0.266	15.385%	0.346	+29.03
Heterospecific	0.011	0.004	31.428	**.001**	.034	6.943	**.004**	0.249	9.091%	0.409	+58.07

White birch	MHD		PT‐MHD		PERMANOVA			Hypervolume	Niche volume metrics		
Between ER	**NP**	**CF**	*F* value	*p*‐Value	*R* ^2^	*F* statistic	*p*‐Value	Jaccard	Overlap	Nestedness	ITV (%)
	0.014	0.010	9.174	**.005**	.251	75.999	**.001**	0.163	5.166%	0.067	+46.65
CT: across ecoregions	**Con**	**Hetero**	*F* value	*p*‐Value	*R* ^2^	*F* statistic	*p*‐Value	Jaccard	Overlap	Nestedness	ITV
	0.013	0.015	0.731	.404	.018	4.075	**.021**	0.552	23.718%	0.623	+68.92
CT: within ecoregions	**Con**	**Hetero**	*F* value	*p*‐Value	*R* ^2^	*F* statistic	*p*‐Value	Jaccard	Overlap	Nestedness	ITV
Northern Peninsula	0.012	0.014	0.120	.750	.003	0.480	.577	0.534	0.457%	0.995	+66.48
Central Forest	0.012	0.008	5.495	**.017**	.117	9.163	**.001**	0.334	43.396%	0.127	−3.66
CT: between ecoregions	**NP**	**CF**	*F* value	*p*‐Value	*R* ^2^	*F* statistic	*p*‐Value	Jaccard	Overlap	Nestedness	ITV
Conspecific	0.012	0.012	0.068	.803	.236	8.038	**.001**	0.132	0.000%	0.000	−13.12
Heterospecific	0.014	0.008	13.415	**.001**	.257	68.702	**.001**	0.093	2.449%	0.079	+57.02

*Note*: Results for balsam fir and white birch are separated within the table. The first column describes the level of comparison: Between ecoregion is our species‐level comparison, and community types (CT) are presented for CT: across ecoregion, CT: within ecoregion, and CT: between ecoregions. In the second column, we present the Multivariate Homogeneity test of Dispersion (MHD) values as the average distance to median for each niche; we denote Northern Peninsula and Central Forest ecoregions as NP and CF, respectively; we denote conspecific and heterospecific community types as con and hetero, respectively. In the third column, we present results for Permutation test for Homogeneity of Multivariate Dispersion (PT‐MHD) and report the *F* value and *p*‐value for the niche comparisons of dispersion. In the fourth column, we present Permutational Multivariate Analysis of Variance (PERMANOVA) results and report the *R* ^2^, *F* statistics, and *p*‐value for niche comparisons. In the fifth column, we present our hypervolume similarity assessment and report the Jaccard similarity index. In the sixth column, we report niche metrics of percent overlap (%), nestedness (i.e., varies between 0 and 1, where 0 = no overlap and 1 = a smaller niche occupying space within a larger niche) and ITV as the difference between relative niche volumes. The sign reported in the ITV column indicates if ITV increased (+) or decreased (−) and the following describes how ITV was determined. For species‐level comparisons, we subtracted Central Forest niches from Northern Peninsula niches. For community‐level comparisons across ecoregions, we subtracted conspecific niches from the heterospecific niches. Similarly, for within‐ecoregion comparisons we subtracted conspecific niches from the heterospecific niches for a given ecoregion. For community‐level comparisons of between ecoregions, Central Forest conspecific were subtracted from Northern Peninsula conspecifics and similarly for heterospecific comparisons. Bolded *p*‐value indicates significant results where *p* ≤ .05.

**TABLE 3:** Northern and southern ecoregions differences for each foliar elemental trait.

(a) Species level: between ecoregion	Balsam fir			White birch		
Ecoregion	C	N	P	C	N	P
Northern Peninsula	52.122 ± .074	1.029 ± .009	0.125 ± .002	49.836 ± .115	2.784 ± .037	0.282 ± .005
Central Forest	52.327 ± .046	0.865 ± .018	0.076 ± .003	50.381 ± .096	1.641 ± .055	0.155 ± .008
Difference	−.205	.164	.049	−.545	1.143	.127

(b) Community level: between ecoregion	Conspecific			heterospecific		
Balsam fir	C	N	P	C	N	P
Northern Peninsula	52.075 ± .107	1.016 ± .013	0.125 ± .002	52.166 ± .103	1.04 ± .013	0.126 ± .003
Central Forest	52.315 ± .065	0.84 ± .024	0.079 ± .005	52.339 ± .066	0.89 ± .026	0.073 ± .004
Difference	−.240	.176	.046	−.173	.150	.053

(c) Community level: between ecoregion	Conspecific			Heterospecific		
White birch	C	N	P	C	N	P
Northern Peninsula	49.86 ± .548	3.07 ± .179	0.334 ± .021	49.835 ± .118	2.775 ± .037	0.281 ± .005
Central Forest	49.945 ± .192	1.81 ± .117	0.188 ± .014	50.59 ± .095	1.56 ± .055	0.14 ± .008
Difference	−.085	1.260	.146	−.755	1.214	.141

*Note*: Average values with standard errors are presented for each foliar trait: C, N, and P concentrations (%) for species‐level between ecoregions (a) and community level between ecoregions (b/c). Central Forest was subtracted from Northern Peninsula to determine differences in percent foliar elemental traits.

### 3.3 Community level: across ecoregions

**FIGURE 4:**
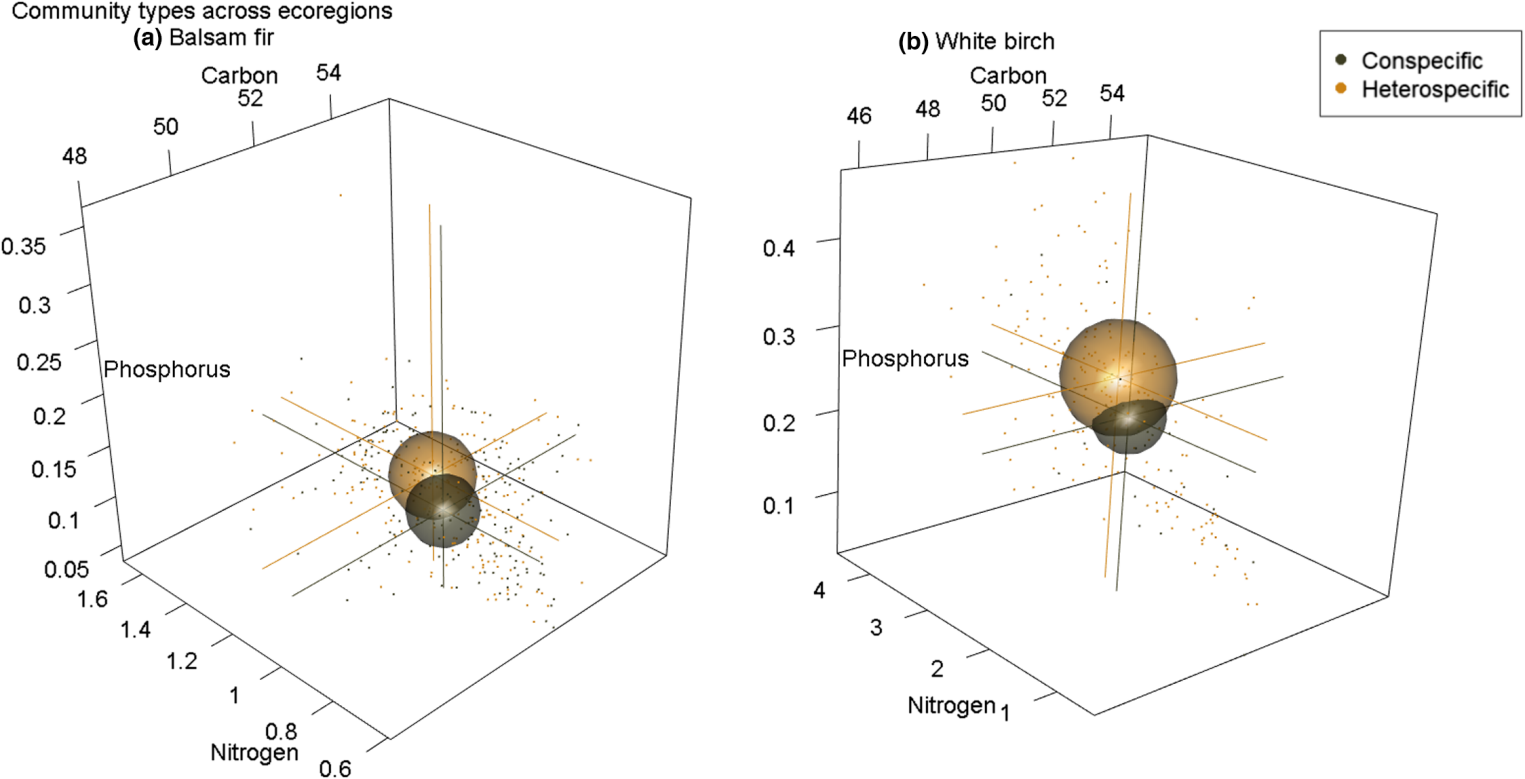
Spherical representations of niche hypervolumes at the community level for across‐ecoregion comparisons for balsam fir (a) and white birch (b). Plot size represents the total stoichiometric volume of C, N, and P for each focal species. Corresponding drop lines to axes indicate the average C, N, and P value for each niche.

We found mixed support for our hypotheses that the heterospecific niche of balsam fir (H2) should be displaced and the heterospecific niche of white birch (H3) should expand in volume relative to their conspecific niche. Our PCA shows heterospecific conditions have a limited effect on balsam fir (Figure 2c). In contrast, we see potential expansion effects for white birch (Figure 2d). PERMANOVA results reaffirm our mixed support as balsam fir conspecific and heterospecific niche hypervolumes were not significantly different (*F* = 0.646, *p‐*value = .458); however, white birch niche hypervolumes were (*F* = 4.075, *p‐*value = .021; Table 2). In addition, non‐significant PT‐MHD and MHD results support PERMANOVA interpretations (Table 2). The Jaccard similarity index was moderately high for both balsam fir (0.709) and white birch (0.552). For balsam fir, our niche volume metrics indicated moderate overlap (43.860%), moderate nestedness (0.276), and increased ITV via niche volume (+35.49%) for the heterospecific niche (see Figure 4a). For white birch, niche volume metrics indicated a low overlap (21.718%), high nestedness (0.623), and increased ITV via niche volume (+68.92%) for the heterospecific niche (see Figure 4b and Table 2). Lastly, our white birch comparison is less reliable via low sample size for the conspecific niche (Appendix S3: Figure S2). As well, see Appendix S6: Figure S4 for a pairwise scatter plot comparison of foliar C, N, and P via conspecific versus heterospecific groups across ecoregions for balsam fir and white birch.

### 3.4 Community level: within ecoregions

**FIGURE 5:**
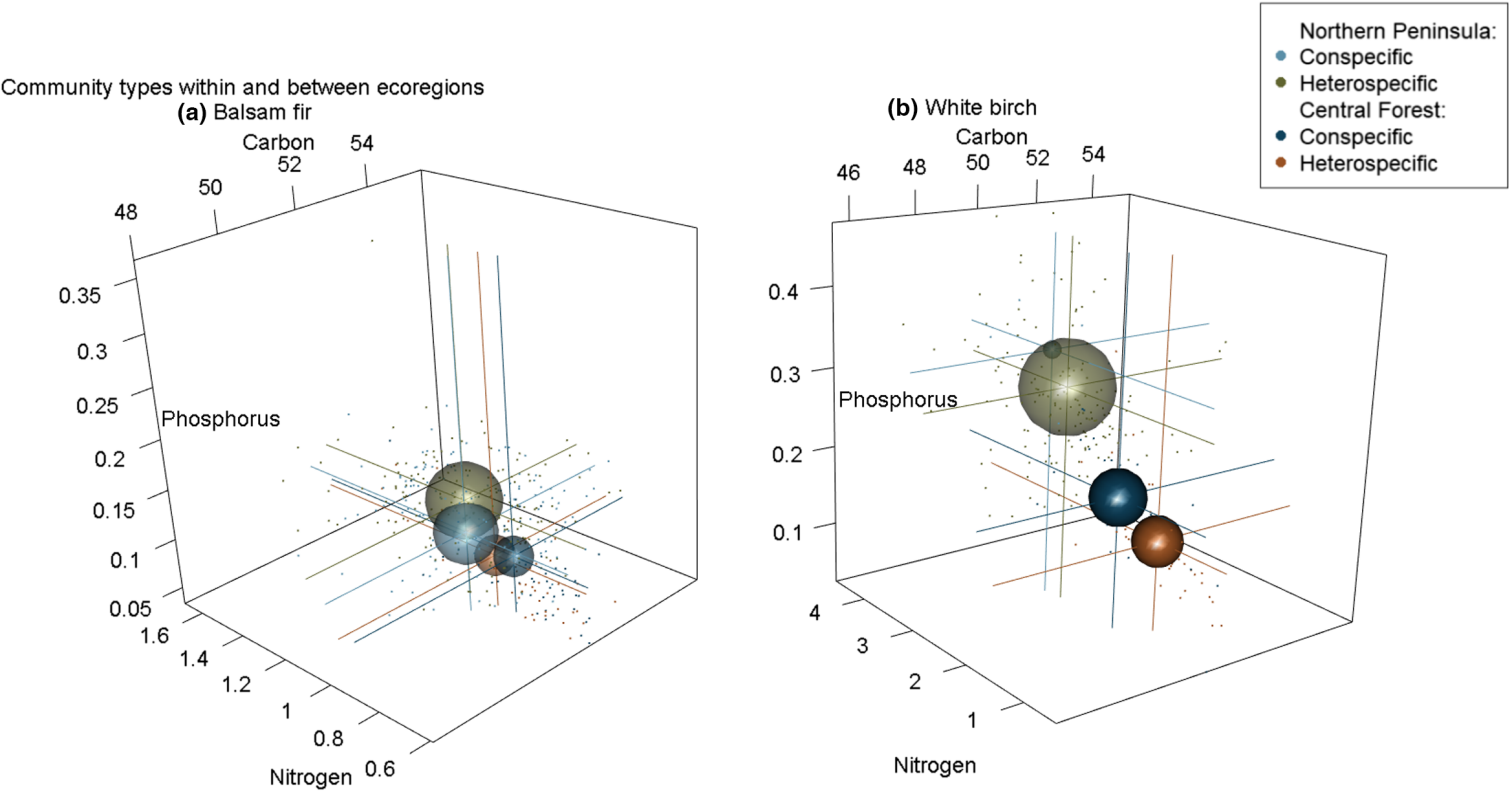
Spherical representations of niche hypervolumes at the community level for within and between‐ecoregion comparisons for balsam fir (a) and white birch (b). Plot size represents the total stoichiometric volume of C, N, and P for each focal species. Corresponding drop lines to axes indicate the average C, N, and P value for each niche. Note that for white birch, the Northern Peninsula conspecific niche is nested completely within the Northern Peninsula heterospecific niche.

We find mixed support for our hypotheses that the heterospecific niche of balsam fir (H4) should be displaced and the heterospecific niche of white birch (H5) should expand in volume relative to their conspecific niche within a given ecoregion. Our PCA showed a high degree of similarity between heterospecific and conspecific niche for balsam fir (Figure 2e). In comparison, we observed a potential expansion effect for white birch heterospecific niche relative to the conspecific niche (Figure 2f). PERMANOVA results reaffirm our mixed support as balsam fir conspecific and heterospecific niche hypervolumes are not significantly different in the Northern Peninsula (*F* = 0.450, *p‐*value = .570), and Central Forest (*F* = 0.306, *p‐*value = .726) ecoregion. For white birch, conspecific and heterospecific niche hypervolumes are not significantly different in the Northern Peninsula ecoregion (*F* = 0.480, *p‐*value = .577); however, these niche hypervolumes are significantly different in the Central Forest ecoregion (*F* = 9.163, *p‐*value = .001; Table 2). Non‐significant PT‐MHD and MHD results support PERMANOVA interpretations (Table 2), except for white birch conspecific and heterospecific niche hypervolume comparisons in the Central Forest (*F* = 5.495, *p‐*value = .017). The Jaccard similarity index was moderately high for both balsam fir in the Northern Peninsula and Central Forest ecoregion (0.672 and 0.566, respectively) and similarly for white birch (0.534, and 0.334, respectively; Table 2). For balsam fir, in both Northern Peninsula and Central Forest ecoregions our niche volume metrics indicate moderate overlap (40.426% and 50%, respectively), moderate‐to‐low nestedness (0.251 and 0, respectively), and increased ITV via heterospecific niche volume in the Northern Peninsula ecoregion (+29.04%) and with no difference in the Central Forest ecoregion (Figure 5a). For white birch, in both Northern Peninsula and Central Forest ecoregions our niche volume metrics indicate low‐to‐moderate overlap (0.457% and 43.396%, respectively), high‐to‐low nestedness (0.995 and 0.127, respectively), and increased ITV via heterospecific niche volume in the Northern Peninsula ecoregion (+66.48%) and decreased in the Central Forest ecoregion (−3.66%; see Figure 5b, Table 2). Lastly, our white birch comparisons are less reliable via low sample sizes for Northern Peninsula conspecific, Central Forest conspecific, and heterospecific niche hypervolumes (Appendix S3: Figure S2). See Appendix S7: Figure S5 for a pairwise scatter plot comparison of foliar C, N, and P via conspecific versus heterospecific groups within and between ecoregions for balsam fir and white birch.

### 3.5 Community level: between ecoregions

Our hypotheses for both balsam fir and white birch that conspecific and heterospecific niches for our northern ecoregion should operate within larger trait space (i.e., niche volume) are supported by our results. Our PCA shows community‐level niches in the Northern Peninsula ecoregion exhibit larger variance than those community niches found in the Central Forest ecoregion (Figure 2e,f). PERMANOVA results reaffirm our hypothesis as significant differences for balsam fir conspecific (*F* = 7.581 and *p*‐value = .005) and heterospecific (*F* = 6.943 and *p*‐value = .004) niche hypervolumes were detected between ecoregions. Similarly, significant differences for white birch conspecific (*F* = 8.038 and *p*‐value = .001) and heterospecific (*F* = 68.702 and *p*‐value = .001) niche hypervolumes were detected between ecoregions (Table 2). Although we determined similar MHD results for our comparisons, we found significant PT‐MHD results for balsam fir conspecific (*F* = 25.902 and *p*‐value = .001) and heterospecific (*F* = 31.428 and *p*‐value = .001) and for white birch heterospecific (*F* = 13.415 and *p*‐value = .001) niche hypervolumes between ecoregions. The Jaccard similarity index was low for balsam fir conspecific (0.266) and heterospecific (0.249) niches between ecoregions, with similar results for white birch conspecific (0.132) and heterospecific (0.093) niches. For balsam fir, both conspecific and heterospecific niches between ecoregions exhibited low overlap (15.385% and 9.091%, respectively), moderate/low nestedness (0.346 and 0.409, respectively), and increased ITV via niche volume in the Northern Peninsula ecoregion (+29.04% and +58.07%, respectively; see Figure 5a). For white birch, both conspecific and heterospecific niches between ecoregions exhibited low overlap (0% and 2.449%, respectively), low nestedness (0 and 0.079, respectively), and increased ITV via northern heterospecific niches (+57.02%) and decreased northern conspecific niches (−13.12%; Figure 5b). In addition, balsam fir foliar N and P were greater for the Northern Peninsula ecoregion for both conspecific (0.176% and 0.046%, respectively) and heterospecific niche hypervolumes (0.15% and 0.053%, respectively), while foliar C was higher in the Central Forest ecoregion for both conspecific (0.24%) and heterospecific niche hypervolumes (0.173%; Table 3b). White birch foliar N and P were greater for the Northern Peninsula ecoregion for both conspecific (1.26% and 0.146%, respectively) and heterospecific niche hypervolumes (1.214% and 0.141%, respectively), while foliar C was higher in the Central Forest ecoregion for both conspecific (0.085%) and heterospecific niche hypervolumes (0.755%) (Table 3b). Lastly, our white birch comparisons are less reliable via low sample sizes for Northern Peninsula conspecific, Central Forest conspecific and heterospecific niche hypervolumes (Appendix S3: Figure S2). See Appendix S7: Figure S5 for a pairwise scatter plot comparison of foliar C, N, and P via conspecific versus heterospecific groups within and between ecoregions for balsam fir and white birch.

## 4 DISCUSSION

Constructing niche hypervolumes using axes of foliar C, N, and P traits allows us to relate variability in species resource strategies to different environmental conditions. In this study, we advance the application of the elemental niche to describe species differences in response to environmental conditions (see González et al., 2017; He et al., 2019; Peñuelas et al., 2019; Sardans et al., 2021). Specifically, we focus on the species level by comparing foliar elemental niche hypervolumes between ecoregions and at the community level by comparing conspecific and heterospecific niche hypervolumes across, within, and between ecoregions. We find evidence to support (H1) that at a species level both balsam fir and white birch exhibit larger elemental niche hypervolumes that are statistically different between ecoregions. At a community level, between ecoregions, we find no support for balsam fir (H2) niche hypervolume displacement patterns; however, we do find evidence to support white birch (H3) niche hypervolume expansion. At a community level within ecoregions, we find no evidence to support balsam fir (H4) niche hypervolume displacement or white birch (H5) niche hypervolume expansion patterns in the Northern Peninsula ecoregion; however, we do find statistical support for white birch niche hypervolume expansion in the Central Forest ecoregion. Lastly, at the community level between ecoregions, we find evidence to support (H6) that conspecific and heterospecific niche hypervolumes are statistically different for both species. Our results suggest that elemental niche differences for our focal species largely occur in response to broad‐scale biophysical conditions with minimal effects at the local community scale.

### 4.1 Biogeographical niche patterns

As expected, individuals from our northern ecoregion contain greater amounts of N and P and exhibited wider elemental niche plasticity compared with their southern counterparts for species‐level (H1) and community‐level (H6) comparisons (Figures 2, 3, 5). Ecoregions are distinguished by their biophysical properties, which include major physiographic and minor macroclimatic differences (Ecological Stratification Working Group, 1996). The mean annual summer and winter temperatures between the Northern Peninsula and Central Forest ecoregion differ by 1.5 and 1°C, respectively. These differences likely contribute to the increased N, P, and elemental niche plasticity we observed in our focal species. The effects of temperature on plant growth rates and underlying biochemical/physiological processes are well documented (Gillooly et al., 2001). Indeed, several studies have shown how a 2–5°C temperature decrease can result in a 3% increase in N and P in plants and this aligns well with our results (Table 3; for synthesis see Woods et al., 2003). Furthermore, our results provide support for the temperature‐plant physiology hypothesis (Reich & Oleksyn, 2004); plants at higher latitudes in colder environments contain greater amounts of N and P.

Moreover, although we did not compare foliar elemental niche differences between our focal species with respect to ecoregions, there are general patterns of note. Balsam fir and white birch occupy different C, N, and P trait space at a species‐level (Figure 3a,b) and community‐level between ecoregions (Figure 5a,b). Across these scales, balsam fir foliar C, N, P is tightly clustered compared with white birch where foliar C, N, and P are highly plastic (density contours from pairwise trait comparisons show similar patterns of trait plasticity; see Appendix S6: Figure S4 and Appendix S7: Figure S5). Our focal species have different geographic distributions (see Appendix S1: Figure S1 for species distribution maps). Thus, the variability of foliar C, N, and P niche breadth may relate to their biological tolerances of temperature changes across the variable environments of their geographic range (i.e., stenothermal vs. eurythermal species; van Dijk et al., 1999). Foliar elemental niche differences or changes in C, N, and P variability may provide linkages to describe the realized niche of species in response to different environmental conditions species experience across their geographic range (fundamental niche; Carscadden et al., 2020). Future studies may consider how local interspecific niche variability differs across a species geographic distribution and how this in turn contributes to our understanding of trait variability and niche breadth.

Furthermore, these results allow us to generalize how the forage of our focal species contributes to dynamics at higher trophic levels and ecosystem processes. Moose on the island of Newfoundland preferentially browse juvenile balsam fir and white birch (Dodds, 1960). In different ecoregions, differing N and P forage quality may translate to different rates of browsing and nutritional condition of moose with implications for population dynamics and space‐use foraging decisions (Hoy et al., 2021). Moreover, over space and time, differing foliar N and P contributions to litter quality via leaf senescence and herbivore fecal depositions will likely influence biogeochemical processes and feedbacks (Shen et al., 2011). These linkages to ecosystem processes provide a functional picture of how the ebb and flow of N and P influence the ecology of landscape via spatial flows of N and P through herbivory, leaf litter contributions, and dissolved nutrients in hydrological systems.

### 4.2 Community‐level niche patterns

Although we expected to reveal heterospecific niche patterns of displacement (H2/H4) for balsam fir using a principal components analysis coupled with a PERMANOVA test, we did not observe statistical significance for these patterns. For instance, conspecific and heterospecific niches of balsam fir across‐ and within‐ecoregion comparisons differed only slightly (Figure 2c,e). This suggests that balsam fir likely maintains a highly rigorous elemental homeostasis regardless of community‐level conditions. However, between‐ecoregion comparisons show that these community‐level niches operate in different elemental trait space. Thus, under elementally different community‐litter‐nutrient scenarios, trade‐offs are likely made between growth, reproduction, and survival that balance the allocation of C, N, and P to maintain a foliar elemental equivalence that is reflective of large‐scale biogeographical conditions (Dumais & Prevost, 2014). As well, white birch sheds its foliar material annually, with differential litter contributions depending on the amount and size of birch present. This may provide an adequate supply of N and P coupled with early season retrieval that allows balsam fir to maintain an elemental equivalence in heterospecific communities (Giordano, 2013; Persson et al., 2010). Alternatively, other local factors not considered in this study, such as light and topographic position, may be important drivers of foliar C, N, and P (Macek et al., 2019). Moreover, across eastern boreal landscapes, the occurrence of balsam fir and white birch in pure and mixedwood stands can be used to represent patches (i.e., coniferous, deciduous, and mixedwood patches; see Hansson, 1992; Pastor et al., 1999). Thus, our results highlight how emergent stand‐scale patterns of resource quality in terms of forage may inform landscape patterns. For instance, if balsam fir remains elementally similar across these differing community types, this provides an invariant parameter to characterize animal foraging behaviors (Duparc et al., 2020) and consequences of animal vectored energy and matter transfers across spatial scales (Dézerald et al., 2018).

In comparison, we expected white birch to exhibit a niche expansion pattern for heterospecific conditions relative to their corresponding conspecific niche at the species level (H3) and community level (H5). Although we did observe a significant niche hypervolume expansion pattern at the species level, at the community‐level heterospecific niches contracted, including a significant contraction for the Central Forest ecoregion. This was unexpected. We hypothesized white birch would exhibit greater elemental plasticity under heterospecific community types regardless of spatial extent. Yet, we observe two different types of heterospecific niche hypervolume responses depending on spatial scale. As well, the ITV differed between our species and community‐level comparisons. Furthermore, we suspect the low sample size of our Northern Peninsula ecoregion niche produced an artificial increase given the high niche overlap and nestedness between conspecific and heterospecific niche hypervolumes.

Overall, our results suggest that white birch foliar C, N, and P are likely influenced by both regional (biogeographical) and localized conditions (Cornell & Lawton, 1992; Lu et al., 2011). For instance, balsam fir produces durable, long‐lived, lignified foliar tissue with limited seasonal litter contributions of recalcitrant material, which is known to reduce soil decomposition rates (Bardgett et al., 1998), alter microbial community structure, and change nutrient pathways (Hobbie, 2015). Thus, recalcitrant litter contributions may reduce white birch nutrient retrieval and N/P use‐efficiencies and produce the niche hypervolume contraction patterns we observed (Figure 2f; He et al., 2010; Krishna & Mohan, 2017). Moreover, our focal species differ in terms of their palatability. For instance, balsam fir exhibits a constant chemical defense profile while white birch exhibits compensatory strategies of allocating N and P to phytochemical production in response to herbivory (Bennett & Wallsgrove, 1994). In heterospecific patches, palatable species, such as white birch, likely experience greater top‐down pressure (Agrawal et al., 2006). Under these heterospecific community conditions, the interaction of nutrient availability (Coley et al., 1985) and herbivory (Daufresne & Loreau, 2001) events may elicit a reduction in white birch foliar C, N, and P. As well, white birch can behave similar to a clonal species when mycorrhizal relationships are present and can allocate resources through root connections to other individuals (Deslippe & Simard, 2011). Thus, differing litter‐nutrient input conditions, herbivorous interactions due to palatability, and the extent of mycorrhizal connections, may collectively influence the foliar C, N, and P of white birch and explain the different niche patterns we observed at the species and community level (Figure 2d,f).

### 4.3 Study limitations

Our study compares data from two research projects with differing sampling designs and as such there are certain limitations to consider when interpreting our results. First, although we collected data/foliar samples in a similar way between these two projects, there are differences in terms of the spatial distribution of sample plots that may have influenced the spatial autocorrelation of samples and thus our interpretation of the findings. However, the two projects do target similar forest units: coniferous, deciduous, and mixedwood forest stands across a range of representative age classes. Second, our sampling of foliar material occurred in two different years with the Northern Peninsula sampled in 2015 and the Central Forest sampled in 2016. Despite the potential for temporal differences in foliar C, N, and P between these ecoregions, we suspect the observed effect is due to biogeographical differences. In 2017, we resampled balsam fir and white birch foliar C, N, and P in the Central Forest ecoregion at the same sample sites. Using 2017 foliar C, N, and P, we constructed conspecific and heterospecific niche hypervolumes and compared them with 2016 conspecific and heterospecific niche hypervolumes. We tested these temporal foliar elemental niche hypervolumes using the same approach described above. Where PERMANOVA results differed significantly for balsam fir 2016 (*n* = 95) and 2017 (*n* = 30) and white birch 2016 (*n* = 71) and 2017 (*n* = 41) temporal foliar elemental niche hypervolumes, PT‐MHD also differed significantly. Thus, we are unable to rely on PERMANOVA results (see Appendix S8: Figure S6 for PCA; Appendix S9: Figure S7 for spherical niche hypervolumes; Appendix S10: Figure S8 for scatter plot kernel density comparisons; and Appendices S11–S13: Table S3–S5 for niche hypervolume sample size, statistical summary, and PERMANOVA results, respectively). Overall, given our temporal comparisons, we suspect the effect observed in this study is likely due to biogeographical differences (for temporal comparisons of foliar stoichiometric traits see Richmond et al., 2021).

The inference for some of our comparisons is likely hindered due to small and unbalanced sample sizes, which may influence trait data dispersion patterns and the output of PERMANOVA tests (Mcardle & Anderson, 2001). To assess this limitation, we subsampled an increasing number of individuals at specified intervals and calculated 999 randomized permutations and 95% confidence of niche hypervolumes at each interval (see Appendix S3: Figure S2). In the Results section, we highlight these less reliable niche hypervolumes. These include white birch conspecifics across ecoregions (*n* = 28), Northern Peninsula conspecific (*n* = 5), and Central Forest conspecific (*n* = 21) and heterospecific niche hypervolumes (*n* = 48). These niches do not saturate, and as such, comparisons using these niche hypervolumes are less reliable. More importantly, our sample size analysis demonstrates a threshold requirement of sample sizes needed to test for foliar elemental niche differences. As such, our work could help guide future research projects aimed at investigating environmental drivers of foliar niche variability across spatial scales by ensuring they have sufficient sample sizes. Lastly, given that we only control for environmental variability at the ecoregion level or community level and a whole suite of interacting conditions may influence the foliar elemental niches of species, we are unsure if our statistical relevance provides meaningful biological relevance. Future work may consider how certain environmental factors influence the foliar niches of these species between and within ecoregions.

## 5 CONCLUSION

Organisms are collections of elements, predominately C, N, and P (Kaspari & Powers, 2016). For plants, C, N, and P are interconnected and needed in sufficient proportions for proper physiological functioning (Sterner & Elser, 2002). Life history and leaf attributes determine foliar intraspecific variability of C, N, and P traits (Sardans et al., 2021). A species' elemental homeostasis and stoichiometric plasticity constrain an individual's eco‐physiological response and tolerance to differing environmental conditions (Asner et al., 2016; Peñuelas et al., 2019). Thus, constructing niche hypervolumes using dimensions of C, N, and P resource axes allows us to assess how plants respond to different environmental conditions revealing differences in resource acquisition and use (Fajardo & Siefert, 2018; González et al., 2017). Although there are numerous ways to construct and assess niche hypervolumes, our work compliments existing work that explicitly uses a plant elemental/stoichiometric framework (González et al., 2017; Peñuelas et al., 2019; Urbina et al., 2017). Here, we investigate elemental niches between ecoregions at the species level, and across, within, and between ecoregions at the community level. At the species level, we find large‐scale biophysical signals that elemental niches are specific to biogeographical conditions and that our focal species operate within a larger trait space in our northern ecoregion. Our results provide evidence to support eco‐physiological patterns in response to biogeographic differences that are consistent with temperature‐physiological effects on plants (Reich & Oleksyn, 2004). This geographic specificity suggests that species may exhibit elemental homeostatic conditions that are constrained by biogeographical properties. Our findings suggest that using foliar elemental traits from one biogeographic area to predict their condition in another area using similar environmental parameters may yield erroneous results given species‐specific differences to temperature/precipitation conditions (van Dijk et al., 1999; Woods et al., 2003). Moreover, studies aimed at predicting global trends via elemental niches or foliar elemental traits should be cautious about the strength of local effects (Butler et al., 2017). At the community level, we find species‐specific responses to heterospecific conditions for both balsam fir and white birch; however, the patterns observed differed from our predictions and in most cases were statistically insignificant. In general, we found that balsam fir maintains a rigorous elemental homeostasis under heterospecific conditions. These results allow us to form generalizations about the tolerances of coniferous/conservative strategy species and how they use and allocate resources in different biogeographical locations and under different community type scenarios. In comparison, white birch did not exhibit a consistent response to heterospecific conditions with an expansion pattern observed across ecoregions and a contraction pattern observed within and between ecoregions. These results suggest other mechanisms across spatial scales likely influence how white birch uses and allocates elemental resources such as the spatial variability of mycorrhizal relationships (Simard, 2009). As well, sample size issues limit the reliability of white birch niche hypervolume comparisons at the community level and subsequent interpretations of those results. In this study, we did not examine ecoregion‐specific or common environmental factors between ecoregions, which may drive differences in foliar elemental niches. For instance, the different parent material, soil type, and soil texture in these ecoregions may influence the availability of nutrients for uptake via soil pH ranges (Finlay, 1995). In addition, the historical disturbance ecology of an ecoregion, or even localized disturbance events, can have long legacy effects that determine nutrient hot spots and community structure (Korell et al., 2017). Thus, since our study only partially explained some of the variability in foliar elemental concentrations, other environmental factors may be more biologically relevant. Future work may consider how differing environmental gradients such as soil structure, disturbance history, ontogeny, and finer resolutions of community composition (including species dominance effects related to the biomass‐ratio hypothesis) may influence the elemental niche of species (Tardif et al., 2014).

## AUTHOR CONTRIBUTIONS

**Travis R. Heckford:** Conceptualization (lead); data curation (equal); formal analysis (lead); investigation (lead); methodology (lead); writing – original draft (lead); writing – review and editing (lead). **Shawn J. Leroux:** Conceptualization (equal); data curation (equal); formal analysis (equal); funding acquisition (equal); investigation (equal); methodology (equal); writing – review and editing (equal). **Eric Vander Wal:** Conceptualization (equal); data curation (equal); formal analysis (equal); funding acquisition (equal); investigation (equal); methodology (equal); writing – review and editing (equal). **Matteo Rizzuto:** Conceptualization (equal); formal analysis (equal); investigation (equal); methodology (equal); writing – review and editing (equal). **Juliana Balluffi‐Fry:** Conceptualization (equal); formal analysis (equal); investigation (equal); methodology (equal); writing – review and editing (equal). **Isabella C. Richmond:** Conceptualization (equal); formal analysis (equal); investigation (equal); methodology (equal); writing – review and editing (equal). **Yolanda F. Wiersma:** Conceptualization (equal); data curation (equal); formal analysis (equal); funding acquisition (equal); investigation (equal); methodology (equal); writing – review and editing (equal).

## CONFLICT OF INTEREST

None declared.

### OPEN RESEARCH BADGES

This article has earned an Open Data badge for making publicly available the digitally‐shareable data necessary to reproduce the reported results. The data is available at https://doi.org/10.6084/m9.figshare.8247134.v1.

## Supporting information


Appendix S1

## Data Availability

All data and code used in the analyses are available via a Dryad repository at: https://doi.org/10.5061/dryad.bk3j9kdg0.
