# Supplementary material for: Ecoregion and community structure influences on the foliar elemental niche of balsam fir (Abies balsamea (L.) Mill.) and white birch (Betula papyrifera Marshall)
Source: Ecol Evol. 2022 Sep 11;12(9):e9244. doi: 10.1002/ece3.9244 (PMC9465200; doi:10.1002/ece3.9244)
Supplement: Supplementary file 1 — Appendix S1 [file ECE3-12-e9244-s001.docx]

# Appendices

## Appendix S1 Fig. S1.


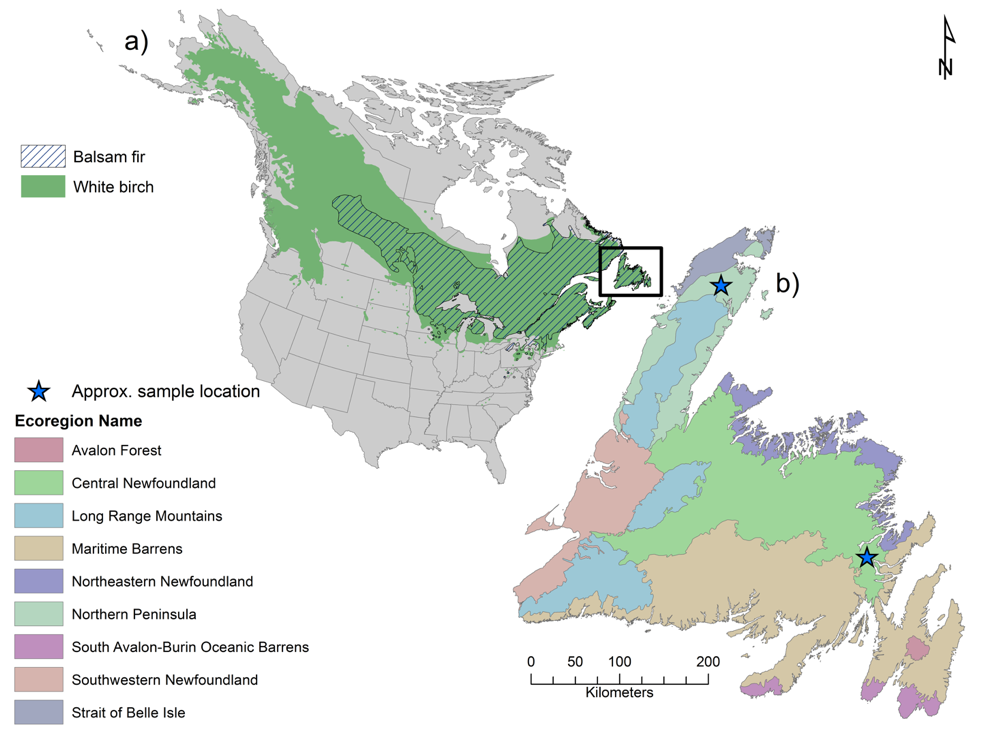


Fig. S1. Study maps showing the (a) geographic distribution of our focal species balsam fir and white birch across North America (Prasad & Iverson, 2003). We sampled balsam fir and white birch individuals from (b) the Island of Newfoundland, at locations indicated by stars in the Northern Peninsula and Central Forest ecoregions.

## Appendix S2 Table S1.

Table S1. PERMANOVA results for each of our niche comparisons for balsam fir and white birch. Our ecoregion comparison of Northern Peninsula (NP) and Central Forest (CF) niches are presented first. Followed by across, within and between ecoregion comparisons of comparisons of conspecific (con) and heterospecific (hetero) niches. Conspecific and heterospecific niche comparisons between our Northern Peninsula and Central Forest populations are present last. Significant *p*-values are bolded.

|  | Balsam fir | | | | |  | White birch | | | | |
| --- | --- | --- | --- | --- | --- | --- | --- | --- | --- | --- | --- |
| Between ecoregions | Df | SS | R^2^ | *F* | *p*-value |  | Df | SS | R^2^ | *F* | *p*-value |
| NP vs CF | 1 | 0.002 | 0.0362 | 14.5919 | **0.001** |  | 1 | 0.0185 | 0.2508 | 75.9986 | **0.001** |
| Residual | 388 | 0.0535 | 0.9638 |  |  |  | 227 | 0.0554 | 0.7492 |  |  |
| Total | 389 | 0.0556 | 1 |  |  |  | 228 | 0.0739 | 1 |  |  |
|  |  |  |  |  |  |  |  |  |  |  |  |
| Across ecoregions |  |  |  |  |  |  |  |  |  |  |  |
| Con vs Hetero | 1 | 1.00E-04 | 0.0017 | 0.6457 | 0.458 |  | 1 | 0.0013 | 0.0176 | 4.0752 | **0.021** |
| Residual | 388 | 0.0555 | 0.9983 |  |  |  | 227 | 0.0726 | 0.9824 |  |  |
| Total | 389 | 0.0556 | 1 |  |  |  | 228 | 0.0739 | 1 |  |  |
|  |  |  |  |  |  |  |  |  |  |  |  |
| Within ecoregion |  |  |  |  |  |  |  |  |  |  |  |
| NP: Con vs Hetero | 1 | 1.00E-04 | 0.0015 | 0.4501 | 0.57 |  | 1 | 1.00E-04 | 0.0031 | 0.4798 | 0.577 |
| Residual | 293 | 0.0505 | 0.9985 |  |  |  | 156 | 0.0456 | 0.9969 |  |  |
| Total | 294 | 0.0506 | 1 |  |  |  | 157 | 0.0458 | 1 |  |  |
|  |  |  |  |  |  |  |  |  |  |  |  |
| Within ecoregion |  |  |  |  |  |  |  |  |  |  |  |
| CF: Con vs Hetero | 1 | 0 | 0.0033 | 0.3055 | 0.726 |  | 1 | 0.0011 | 0.1172 | 9.1626 | **0.001** |
| Residual | 93 | 0.0029 | 0.9967 |  |  |  | 69 | 0.0085 | 0.8828 |  |  |
| Total | 94 | 0.0029 | 1 |  |  |  | 70 | 0.0096 | 1 |  |  |
|  |  |  |  |  |  |  |  |  |  |  |  |
| Con: between ecoregion | 1 | 0.001 | 0.039 | 7.5813 | **0.005** |  | 1 | 0.0015 | 0.2362 | 8.0383 | **0.001** |
| Residual | 187 | 0.0254 | 0.961 |  |  |  | 26 | 0.0049 | 0.7638 |  |  |
| Total | 188 | 0.0264 | 1 |  |  |  | 27 | 0.0065 | 1 |  |  |
|  |  |  |  |  |  |  |  |  |  |  |  |
| Hetero: between ecoregion | 1 | 0.001 | 0.0337 | 6.9427 | **0.004** |  | 1 | 0.017 | 0.2566 | 68.7019 | **0.001** |
| Residual | 199 | 0.028 | 0.9663 |  |  |  | 199 | 0.0492 | 0.7434 |  |  |
| Total | 200 | 0.029 | 1 |  |  |  | 200 | 0.0661 | 1 |  |  |

## Appendix S3 Fig. S2.


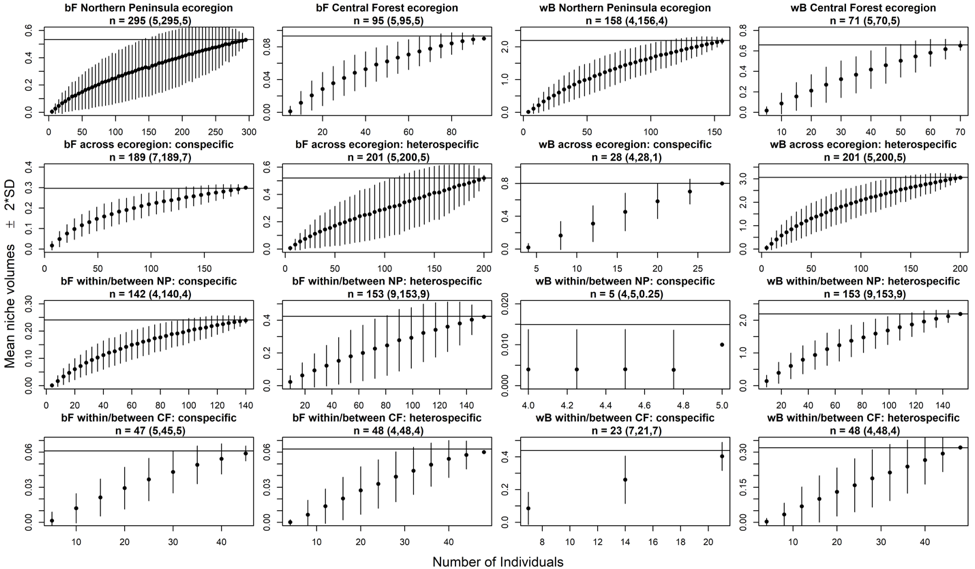


Fig. S2. The effect of increasing sample size on determining niche hypervolume. Results for species level comparisons across ecoregions are shown in the top row of plots (H1). The second row of plots show results for community level comparisons across ecoregions (H2/H3) and the third and fourth row of plots show results for within (H4/H5) and between (H6) comparisons across ecoregions. For each plot we provide the total sample size (n) and the maximum number of samples used to estimate niche hypervolume using 999 randomized permutations at a specified interval of increasing individuals used (i.e., 5, 295, 5 from the first plot indicates a starting samples size of 5, an end sample size of 295, at an interval of 5). The range of variation in our randomized niche volumes was quantified using 95% confidence interval (2 standard deviations from the randomized niche mean). Horizontal lines indicate the total niche hypervolume computed for a given niche hypervolume (i.e., ecoregion, conspecific, heterospecific).

## Appendix S4 Table S2.

Table S2. The sample size for each niche (n), the MVW and *p*-value results of a Shapiro-Wilk test for multivariate normality, here a significant *p*-value indicates these data are not normally distributed. To perform a PERMANOVA data must exhibit a non-normal structure. We further investigate this with Levene’s test for multivariate homogeneity of variance for each of our niche comparisons. This aids in our interpretation of PERMANOVA results. In addition, we provide niche volume as a percentage, relative to the total available niche space. This was calculated following steps outlined by González et al., 2017. Forward slashes (/) indicate insufficient samples sizes needed for calculation. Calculating the Shapiro-Wilk measure requires a minimum of 12 data points (Shapiro & Wilk, 1965) and niche volume requires a minimum of 2 data points (González et al., 2017).

| Balsam fir | n | Shapiro-Wilk (MVW) | *p*-value | Niche volume (%) |
| --- | --- | --- | --- | --- |
| Northern Peninsula | 295 | 0.942 | **3.42x10^-22^** | 85.49 |
| Central Forest | 95 | 0.942 | **3.41x10^-8^** | 14.52 |
| Across ecoregions: conspecific | 189 | 0.979 | **4.93x10^-5^** | 48.39 |
| Across ecoregions: heterospecific | 201 | 0.942 | **2.98x10^-16^** | 83.88 |
| Northern Peninsula: conspecific | 142 | 0.980 | **0.003** | 38.71 |
| Northern Peninsula: heterospecific | 153 | 0.902 | **4.38x10^-20^** | 67.75 |
| Central Forest: conspecific | 47 | 0.937 | **2.72x10^-4^** | 9.68 |
| Central Forest: heterospecific | 48 | 0.928 | **3.84x10^-5^** | 9.68 |
| White birch | n | Shapiro-Wilk (MVW) | *p*-value | Niche volume (%) |
| Northern Peninsula | 158 | 0.973 | **1.30x10^5^** | 66.78 |
| Central Forest | 71 | 0.958 | **5.67x10^-4^** | 20.13 |
| Across ecoregions: conspecific | 28 | 0.975 | 0.894 | 24.39 |
| Across ecoregions: heterospecific | 201 | 0.977 | **2.78x10^-6^** | 93.31 |
| Northern Peninsula: conspecific | 5 | / | / | 0.30 |
| Northern Peninsula: heterospecific | 153 | 0.972 | **7.80x10^-6^** | 66.78 |
| Central Forest: conspecific | 23 | 0.963 | 0.657 | 13.42 |
| Central Forest: heterospecific | 48 | 0.924 | **1.67x10^-5^** | 9.76 |

## Appendix S5 Fig. S3.


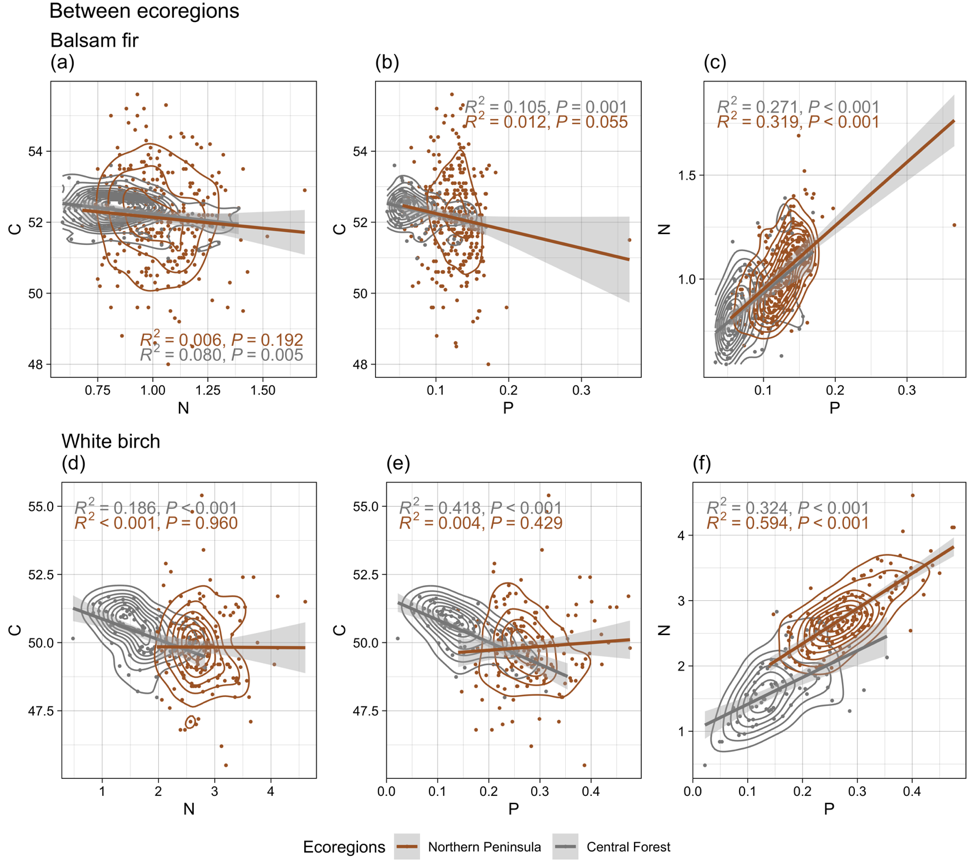


Fig. S3. Species level between ecoregion scatter plots with kernel density estimations presented using Gaussian approximation contour lines for individual pair-wise comparisons of foliar C, N, and P traits for balsam fir (a, b, and c) and white birch (d, e, and f). Colour-coded R^2^ and *p*-values are presented for each trait comparison by ecoregion. This figure complements spherical representations of niches found in in Fig. 3.

## Appendix S6 Fig. S4.


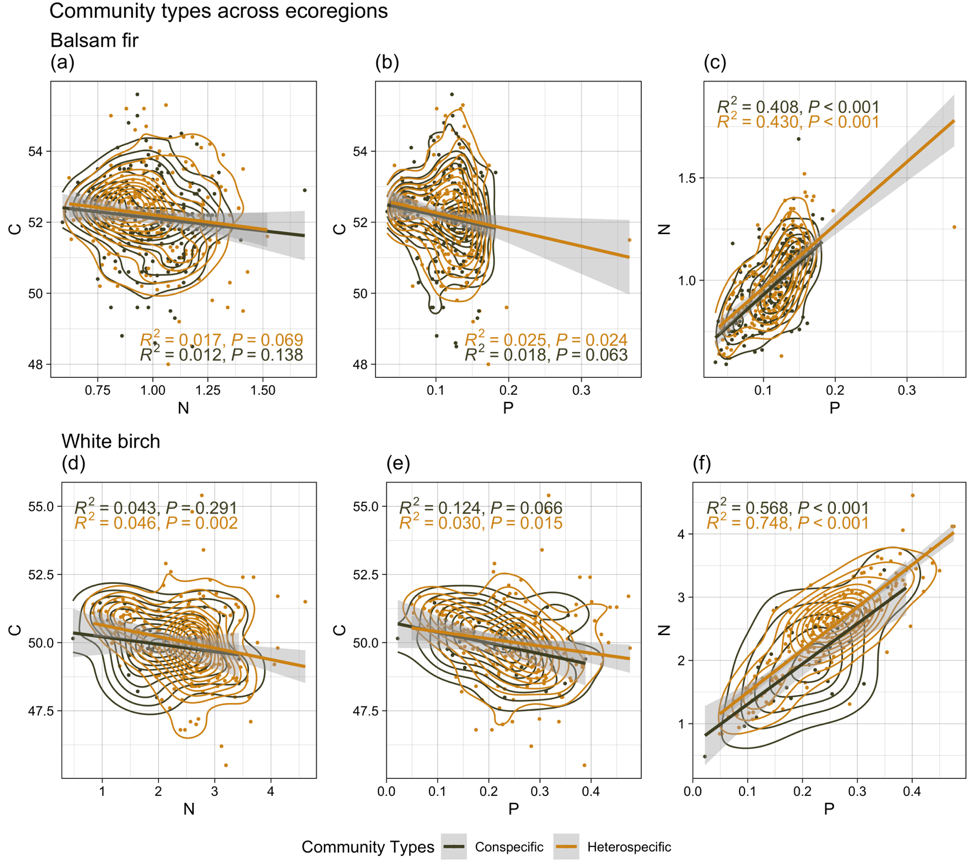


Fig. S4. Community level across ecoregion scatter plots with kernel density estimations presented using Gaussian approximation contour lines for individual pair-wise comparisons of foliar C, N, and P traits for balsam fir (a, b, and c) and white birch (d, e, and f). Colour-coded R^2^ and *p*-values are presented for each trait comparison by ecoregion. This figure complements spherical representations of niches found in Fig. 4.

## Appendix S7 Fig. S5.


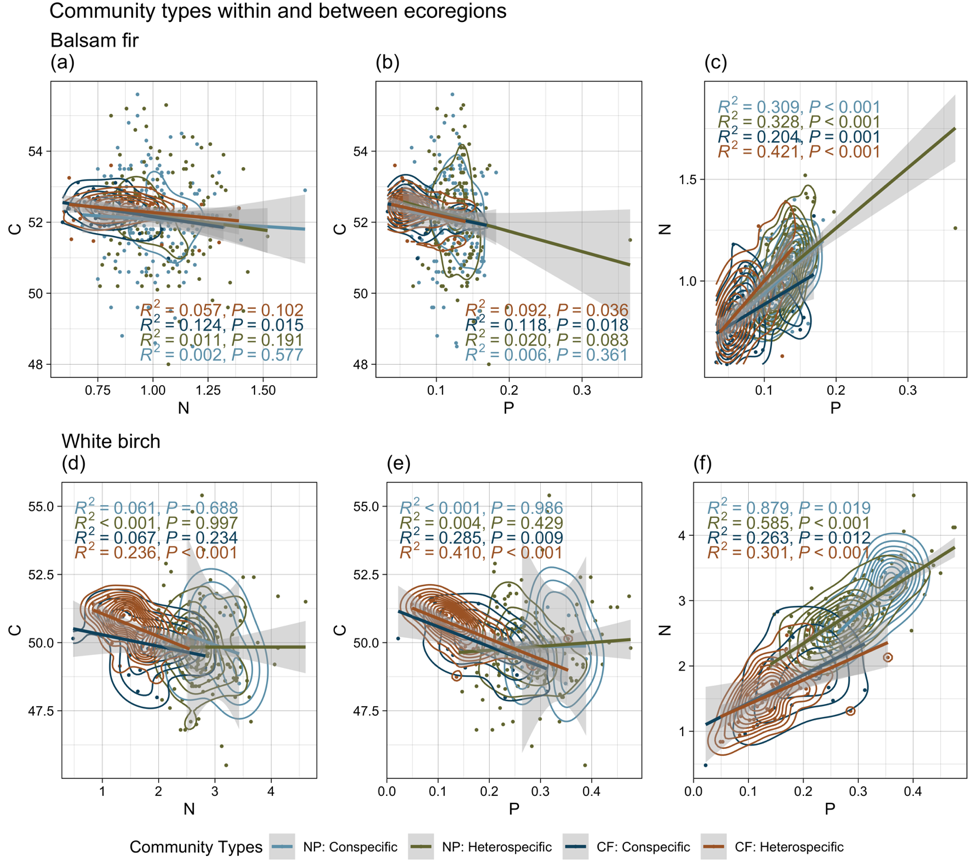


Fig. S5. Community level within and between ecoregion scatter plots with kernel density estimations presented using Gaussian approximation contour lines for individual pair-wise comparisons of foliar C, N, and P traits for balsam fir (a, b, and c) and white birch (d, e, and f). Colour-coded R^2^ and *p*-values are presented for each trait comparison by community type and ecoregion. This figure complements spherical representations of niches found in Fig. 5.

## Appendix S8 Fig. S6.


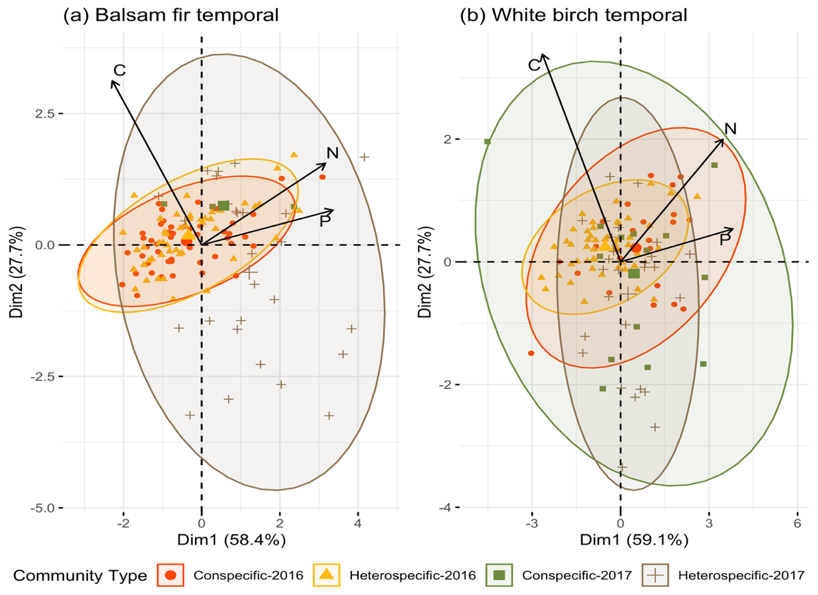


Fig. S6. Principal component analysis (PCA) for our 2016 and 2017 temporal comparison of balsam fir (a) and white birch (b) in the Central Forest population. Ellipses with a 95 % probability are shown for conspecific 2016, heterospecific 2016, conspecific 2017, and heterospecific 2017 niches and coloured as red, yellow, green, and brown respectively. In addition, individuals for these niches are depicted as circles, triangles, squares, and crosshairs, respectively. In both cases the variance is similar for dimension 1 and 2. In all cases N and P highly influence dimension 1 while C influences dimension 2. There was insufficient sample size to construct a balsam fir conspecific 2017 niche.

## Appendix S9 Fig. S7.


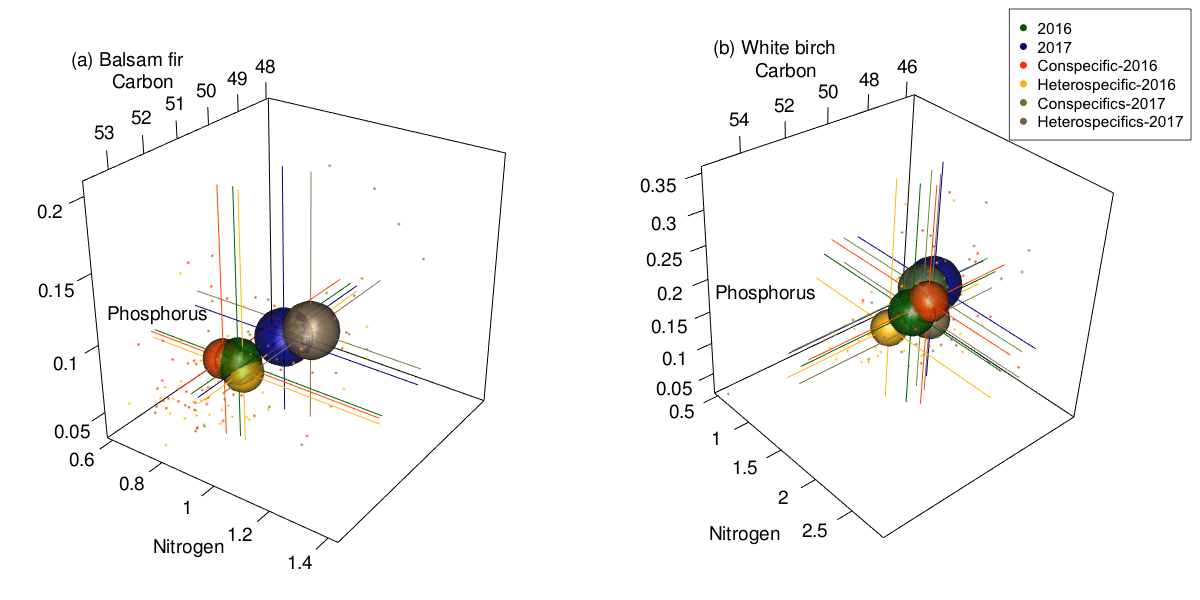


Fig. S7. Spherical representations of temporal foliar C, N, and P niche hypervolumes for balsam fir (a) and white birch (b). For each species we depicted niches as spheres and show average C, N, and P values with droplines for 2016 (n = 95 for fir and n = 71 for birch) and 2017 (n = 30 for fir and n = 41 for birch) niches; 2016 conspecific (n = 47 for fir and n = 23 for birch) and heterospecific (n = 48 for fir and n = 48 for birch) niches; and 2017 conspecific (n = 3 for fir and n = 14 for birch) and heterospecific (n = 27 for fir and n = 27 for birch) niches. Plot size represents the total stoichiometric volume of C, N, and P between years.

## Appendix S10 Fig. S8.


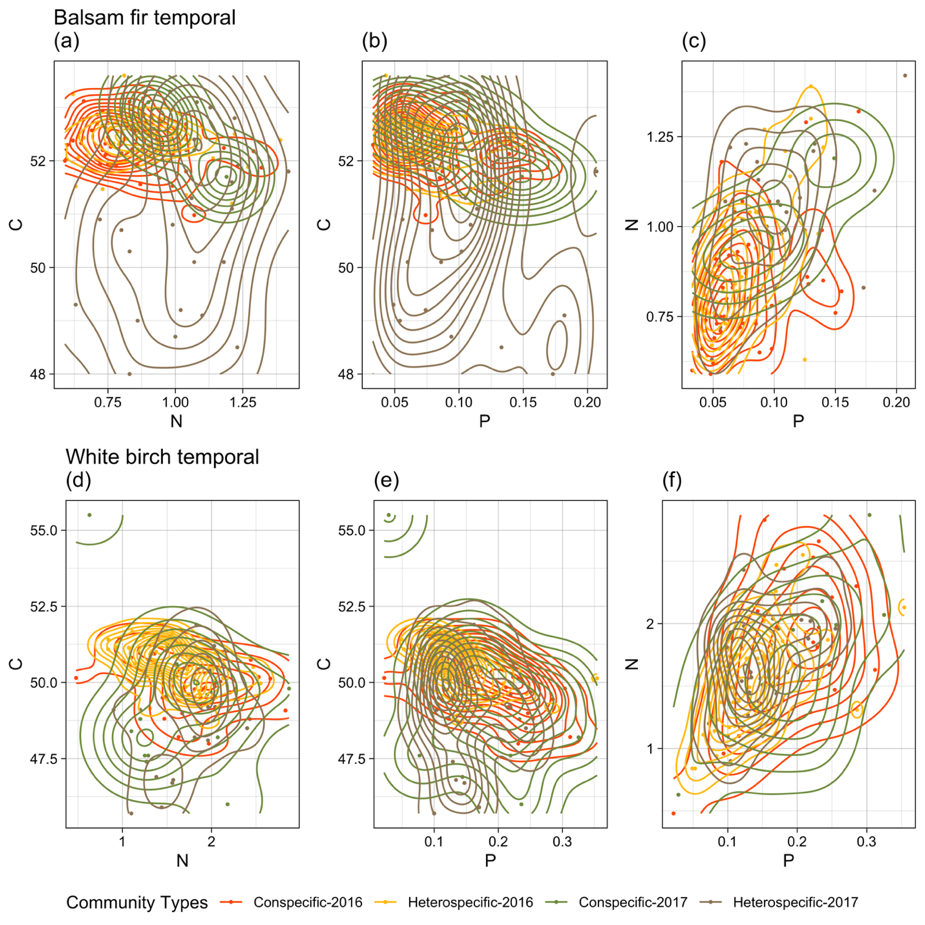


Fig. S8. Scatter plots for our temporal comparisons with kernel density estimations presented using Gaussian approximation contour lines for balsam fir (a, b, and c) and white birch (d, e, and f) showing 2016 and 2017 conspecific and heterospecific groups. This figure complements spherical representations of niches found in Fig. S4.

## Appendix SS11 Table S3.

Table S3. For our temporal comparisons we report the sample size of each niche (n), the MVW and *p*-value results of a Shapiro-Wilk test for multivariate normality, here a significant *p*-value indicates these data are not normally distributed. To perform a PERMANOVA, data must exhibit a non-normal structure. We further investigate this with Levene’s test for multivariate homogeneity of variance for each of our niche comparisons. This aids in our interpretating of PERMANOVA results. In addition, we provide niche volume as a percentage, relative to the total available niche space. This was calculated following steps outlined by González et al., 2017. Forward slashes (/) indicate insufficient samples sizes needed for calculation. Shapiro-Wilk requires a minimum of 12 data points (Shapiro & Wilk, 1965) and niche volume requires a minimum of 2 data points (González et al., 2017). Significant *p*-values are bolded.

| Balsam fir | n | Shapiro-Wilk (MVW) | *p*-value | Niche volume (%) |
| --- | --- | --- | --- | --- |
| 2017 conspecific | 3 | / | / | / |
| 2017 heterospecific | 27 | 0.957 | 0.299 | 61.420 |
| 2016 con/heterospecific | 95 | 0.942 | **3.41x10^-8^** | 29.090 |
| 2017 con/heterospecific | 30 | 0.960 | 0.270 | 61.420 |
| White birch | n | Shapiro-Wilk (MVW) | *p*-value | Niche volume (%) |
| 2017 conspecific | 14 | 0.929 | 0.266 | 47.120 |
| 2017 heterospecific | 27 | 0.972 | 0.838 | 25.280 |
| 2016 con/heterospecific | 71 | 0.958 | **0.001** | 37.920 |
| 2017 con/heterospecific | 41 | 0.968 | 0.269 | 69.520 |

## Appendix S12 Table S4.

Table S4. Summary of temporal niche comparison results for balsam fir and white birch. Results for balsam fir and white birch are separated within the table. The first column indicates the level of comparison, and the second column indicates the year or community type groups being compared for that level. For instance, in the first column we compare groups in the second column at the ecoregion, in this case Central Forest, the community type of conspecific (con) and heterospecific (hetero), or the year level. For the other columns we present the Multivariate Homogeneity test of Dispersion (MHD) for each niche, the Permutation test for Homogeneity of Multivariate Dispersion (PT-MHD) with *F* value and *p*-value for the comparison of dispersion between two niches. As well, we report the Permutational Multivariate Analysis of Variance (PERMANOVA) showing a R^2^, *F* statistic, and *p*-value for each comparison, followed by our hypervolume similarity assessment which reports the Jaccard similarity index. Lastly, we report niche metrics of overlap, nestedness and ITV. The sign of the ITV indicates if ITV increased (+) or decreased (-). For our 2016 and 2017 comparisons in the Central Forest ecoregion, conspecific, and heterospecific niches, ITV is calculated by subtracting niche volumes of 2017 by 2016. For our 2017 comparison of conspecific and heterospecific niches, ITV is calculated by subtracting niche volumes of heterospecific by conspecific. Bolded *p*-value indicate significant results. Forward slashes indicate insufficient samples sizes to compute.

| **Balsam fir** | MHD | | PT-MHD | | PERMANOVA | | | Hypervolume | | Niche volume metrics | | |
| --- | --- | --- | --- | --- | --- | --- | --- | --- | --- | --- | --- | --- |
| **Comparisons** | **2016** | **2017** | F value | *p*-value | R^2^ | F statistic | *p*-value | Jaccard | | Overlap (%) | Nestedness | ITV (%) |
| Central Forest | 0.005 | 0.015 | 87.405 | **0.001** | 0.245 | 40.003 | **0.001** | 0.163 | | 21.739 | 0.167 | + 32.33 |
| Conspecific | 0.005 | 0.006 | 0.583 | 0.458 | 0.019 | 0.936 | 0.363 | 0.329 | | / | / | / |
| Heterospecific | 0.005 | 0.015 | 55.961 | **0.001** | 0.273 | 27.409 | **0.001** | 0.160 | | 13.636 | 0.197 | + 42.02 |
| **Comparisons** | **Con** | **Hetero** | F value | *p*-value | R^2^ | F statistic | *p*-value | Jaccard | | Overlap (%) | Nestedness | ITV (%) |
| 2017 | 0.006 | 0.015 | 3.147 | 0.088 | 0.069 | 2.077 | 0.151 | 0.310 | | / | / | / |
|  |  |  |  |  |  |  |  |  |  |  |  |  |
| **White birch** | MHD | | PT-MHD | | PERMANOVA | | | Hypervolume | | Niche volume metrics | | |
| **Comparisons** | **2016** | **2017** | F value | *p*-value | R^2^ | F statistic | *p*-value | Jaccard | | Overlap (%) | Nestedness | ITV (%) |
| Central Forest | 0.010 | 0.017 | 15.731 | **0.001** | 0.137 | 17.510 | **0.001** | 0.302 | | 37.500 | 0.255 | + 31.6 |
| Conspecific | 0.011 | 0.020 | 4.239 | **0.041** | 0.028 | 0.989 | 0.374 | 0.273 | | 35.484 | 0.245 | + 21.84 |
| Heterospecific | 0.008 | 0.016 | 15.316 | **0.001** | 0.263 | 26.059 | **0.001** | 0.272 | | 18.750 | 0.043 | + 6.89 |
| **Comparisons** | **Con** | **Hetero** | F value | *p*-value | R^2^ | F statistic | *p*-value | Jaccard | | Overlap (%) | Nestedness | ITV (%) |
| 2017 | 0.020 | 0.016 | 0.738 | 0.424 | 0.013 | 0.516 | 0.564 | 0.258 | | 27.273 | 0.170 | - 21.84 |

## Appendix S13 Table S5.

Table S5. PERMANOVA results for each of our temporal niche comparisons with balsam fir in the first and white birch in the second column. Our temporal comparison of conspecific (con) niches is presented first followed by our temporal comparison of heterospecific (hetero) niches. Thirdly, we present a 2017 conspecific versus heterospecific comparison. A comparison for 2016 and 2017 is presented last. Significant *p*-values are bolded. Temporal comparison of conspecific niches for balsam fir is limited due to low sample size (n=3).

|  | Balsam fir | | | | |  | White birch | | | | |
| --- | --- | --- | --- | --- | --- | --- | --- | --- | --- | --- | --- |
|  | Df | SS | R^2^ | *F* | *p*-value |  | Df | SS | R^2^ | *F* | *p*-value |
| Temporal con | 1 | 0 | 0.0191 | 0.9357 | 0.363 |  | 1 | 4.00E-04 | 0.0275 | 0.9888 | 0.374 |
| Residual | 48 | 0.0015 | 0.9809 |  |  |  | 35 | 0.0129 | 0.9725 |  |  |
| Total | 49 | 0.0016 | 1 |  |  |  | 36 | 0.0133 | 1 |  |  |
|  |  |  |  |  |  |  |  |  |  |  |  |
| Temporal hetero | 1 | 0.0034 | 0.273 | 27.409 | **0.001** |  | 1 | 0.005 | 0.2631 | 26.0589 | **0.001** |
| Residual | 73 | 0.0091 | 0.727 |  |  |  | 73 | 0.0141 | 0.7369 |  |  |
| Total | 74 | 0.0125 | 1 |  |  |  | 74 | 0.0191 | 1 |  |  |
|  |  |  |  |  |  |  |  |  |  |  |  |
| 2017 con vs hetero | 1 | 6.00E-04 | 0.069 | 2.0765 | 0.151 |  | 1 | 2.00E-04 | 0.0131 | 0.5161 | 0.564 |
| Residual | 28 | 0.0077 | 0.931 |  |  |  | 39 | 0.0185 | 0.9869 |  |  |
| Total | 29 | 0.0083 | 1 |  |  |  | 40 | 0.0188 | 1 |  |  |
|  |  |  |  |  |  |  |  |  |  |  |  |
| 2016 vs 2017 | 1 | 0.0036 | 0.2454 | 40.0025 | **0.001** |  | 1 | 0.0045 | 0.1373 | 17.5104 | **0.001** |
| Residual | 123 | 0.0112 | 0.7546 |  |  |  | 110 | 0.0284 | 0.8627 |  |  |
| Total | 124 | 0.0149 | 1 |  |  |  | 111 | 0.0329 | 1 |  |  |

References

González, A. L., Dézerald, O., Marquet, P. A., Romero, G. Q., & Srivastava, D. S. (2017). The Multidimensional Stoichiometric Niche. *Frontiers in Ecology and Evolution*, *5*. https://doi.org/10.3389/fevo.2017.00110

Prasad, A. M., & Iverson, L. R. (2003). *Little’s range and FIA importance value database for 135 eastern US tree species* [Map]. https://www.fs.fed.us/nrs/atlas/tree/

Shapiro, S. S., & Wilk, M. B. (1965). An analysis of variance test for normality (complete samples). *Biometrika*, *52*(3–4), 591–611. https://doi.org/10.1093/biomet/52.3-4.591
